# Screening of FDA-Approved Drugs Using a 384-Well Plate-Based Biofilm Platform: The Case of Fingolimod

**DOI:** 10.3390/microorganisms8111834

**Published:** 2020-11-21

**Authors:** Shella Gilbert-Girard, Kirsi Savijoki, Jari Yli-Kauhaluoma, Adyary Fallarero

**Affiliations:** 1 Drug Research Program, Division of Pharmaceutical Biosciences, Faculty of Pharmacy, University of Helsinki, FI-00014 Helsinki, Finland; kirsi.savijoki@helsinki.fi (K.S.); adyary.fallarero@helsinki.fi (A.F.); 2 Drug Research Program, Division of Pharmaceutical Chemistry and Technology, Faculty of Pharmacy, University of Helsinki, FI-00014 Helsinki, Finland; jari.yli-kauhaluoma@helsinki.fi

**Keywords:** fingolimod, biofilm, antibacterial, screening, quorum sensing, *Staphylococcus aureus*, *Acinetobacter baumannii*, *Pseudomonas aeruginosa*

## Abstract

In an effort to find new repurposed antibacterial compounds, we performed the screening of an FDA-approved compounds library against *Staphylococcus aureus* American Type Culture Collection (ATCC) 25923. Compounds were evaluated for their capacity to prevent both planktonic growth and biofilm formation as well as to disrupt pre-formed biofilms. One of the identified initial hits was fingolimod (FTY720), an immunomodulator approved for the treatment of multiple sclerosis, which was then selected for follow-up studies. Fingolimod displayed a potent activity against *S. aureus* and *S. epidermidis* with a minimum inhibitory concentration (MIC) within the range of 12–15 µM at which concentration killing of all the bacteria was confirmed. A time–kill kinetic study revealed that fingolimod started to drastically reduce the viable bacterial count within two hours and we showed that no resistance developed against this compound for up to 20 days. Fingolimod also displayed a high activity against *Acinetobacter baumannii* (MIC 25 µM) as well as a modest activity against *Escherichia coli* and *Pseudomonas aeruginosa*. In addition, fingolimod inhibited quorum sensing in *Chromobacterium violaceum* and might therefore target this signaling pathway in certain Gram-negative bacteria. In conclusion, we present the identification of fingolimod from a compound library and its evaluation as a potential repurposed antibacterial compound.

## 1. Introduction

Infections involving multi-drug resistant bacteria have become extremely harder to treat, as many of the currently used antibiotics are less effective, which has boosted the awareness of the urgent need for new drugs and therapeutic options in recent years [1,2,3]. In addition, bacteria prefer biofilm formation (multicellular communities of cells embedded in a self-produced extracellular matrix composed of polysaccharides, nucleic acids and proteins) as their primary growth mode, which provides the residing cells with higher chemotolerance in comparison to free-floating (planktonic) single cells, thus making infections notoriously hard to treat [4,5]. When adopting a biofilm lifestyle, bacteria change their genome and proteome in ways that contribute to their survival [6,7]. Many cellular mechanisms, involving, e.g., efflux pumps, the downregulation of key metabolic activities and switch to a dormant state, are contributory factors making the cells within the biofilm less susceptible to most antibiotics [8,9]. Biofilm-related infections represent a majority of all bacterial infections and can affect a large variety of organs [5,10]. They are found in up to 60% of chronic wound infections and are particularly prevalent in infections occurring in medical devices, such as orthopedic implants and catheters, as non-biological foreign material offers a ready surface for colonization by single bacteria [11,12,13,14]. Over 50% of all urinary catheters will become infected within two weeks, and up to 2% of all orthopedic alloplastic devices and 4% of heart valves and pacemakers will eventually become infected [15,16]. In those cases, as biofilms cannot be fully destroyed by antibiotic treatments, a chronic infection settles and the device needs to be surgically replaced, a procedure that implicates risks and morbidity [16,17]. Thus, the prevention of biofilm formation is the method of choice to combat device-related infections.

To date, most antibacterial treatments available have been developed to treat acute infections caused by planktonic cells and fail to affect biofilms [18]. In many studies, the activity of new antibacterial compounds is still evaluated exclusively by determining the minimum inhibitory concentration (MIC) of the drug on planktonic cells. Many approaches have been taken to tackle the challenge of antibacterial resistance combined with the natural tolerance of biofilms, including searches for new structures among previously unavailable natural sources and the computer-based design of new molecules. Another promising strategy is to repurpose drugs that have been developed for another therapeutic use into antimicrobials. This approach, while bringing less novelty than screening newly synthesized structures or natural products, has the advantage of increasing the chances of finding biologically active hits. Additionally, compounds that have already been investigated or approved for therapeutic use in humans are more likely to be safe and to have drug-like characteristics than new compounds, reducing the cost and time needed for the development of a drug [19,20].

In the present study, we screened a collection of FDA-approved drugs using a previously optimized 384-well plate-based platform that allows screening the effect of the compounds on both the viability and biomass of the biofilm [21]. The screening was performed against *Staphylococcus aureus*, an important pathogen responsible for a large variety of healthcare-associated infections such as device-related infections, wound infections and sepsis [22]. From the FDA-approved library (containing 774 compounds), 45 compounds were identified as highly effective in preventing the biofilm formation of *S. aureus*. From these, fingolimod, a sphingosine-1-phosphate receptor modulator, was selected for further characterization studies. We explored the ability of this drug to prevent the formation of *S. aureus* biofilms as well as to disrupt pre-formed biofilms. We tested fingolimod on other Gram-positive as well as Gram-negative bacterial species and investigated its mode of action (MoA) via time-kill kinetics, resistance development and quorum sensing (QS) inhibition studies. The antibacterial activity of structural analogues of fingolimod (e.g., sphingosine) has been studied in more detail before, however, to the best of our knowledge, fingolimod’s activity against *S. aureus* was not earlier reported and little is known of its anti-biofilm activity. Taken together, our findings support that fingolimod, a drug registered to treat multiple sclerosis, has an important antimicrobial activity and might serve as an interesting parent structure for chemical optimization efforts to obtain new antibacterial compounds.

## 2. Materials and Methods

### 2.1. Bacterial Strains and Culture Conditions

All bacterial strains, their providers and the analysis performed with each, are shown in Table 1. At the start of all experiments, unless otherwise specified, every strains (except *Pseudomonas aeruginosa* and *Chromobacterium violaceum*) were first grown on a tryptic soy agar (TSA, LAB011, Lab M Ltd., Heywood, UK) plate and incubated overnight at 37 °C under aerobic conditions. Then, the colonies were suspended in 5 mL of tryptic soy broth (TSB, LAB004, Lab M Ltd., Heywood, UK) in a 50 mL Falcon tube (Greiner Bio-One, Kremsmünster, Austria) and grown to exponential phase under aerobic conditions at 37 °C with shaking (220 rpm), until the culture reached a concentration of approximately 1 × 10^8^ colony forming unit (CFU) mL^−1^. The culture was diluted to 1 × 10^6^ CFU mL^−1^ before starting the experiment (or to 7 × 10^4^ CFU mL^−1^ for the screening). The same was done with all *P. aeruginosa* strains using Lennox broth (LB) agar (LBA) (LAB168, Lab M Ltd.) plates and LB (LAB169, Lab M Ltd.) for the liquid culture. For the QS inhibition analysis, *C. violaceum* was grown on LBA overnight at 27 °C and the colonies were used to start the experiment directly.

### 2.2. Compounds

A commercial compound library, the Screen-Well FDA Approved Drug Library Version 2 (ENZO Life Sciences, Helsinki, Finland) containing 774 compounds was used for the screening with the goal of identifying repurposed antibacterial compounds. For follow-up studies, fingolimod, with purity ≥98%, was purchased from Carbosynth (Compton, UK). Fingolimod was dissolved into dimethyl sulfoxide (DMSO) and sonicated for 5 min using a Branson 3800 ultrasonic bath (Cleanosonic, Richmond, VA, USA), at 40 Hz. The antibiotics used in the resistance development experiment (doxycycline (D3447), penicillin G (13752) and oxacillin (28221)) were all bought from Sigma-Aldrich (St. Louis, MO, USA).

### 2.3. Screening of the Compound Library in 384-Well Plates on S. aureus Biofilms

The compounds were diluted in DMSO or sterile H_2_O, according to the provider’s instructions, and tested at a final concentration of 10 µM against *S. aureus* American Type Culture Collection (ATCC) 25923. Cells were diluted in TSB at a concentration of 7.00 × 10^4^ CFU mL^−1^ and grown in polystyrene 384-well plates (Nunc^TM^ 242757, Thermo Fisher Scientific, Waltham, MA, USA) in a volume of 40 µL per well. For the pre-exposure screening, the compounds were added to the wells before the bacterial solution to test the ability of compounds to prevent bacterial growth and plates were incubated for 18 h under aerobic conditions at 37 °C with shaking (220 rpm). For the post-exposure screening, bacteria were grown with no compounds in the same conditions for 18 h, after which the media was changed, and compounds were added on the pre-formed biofilms. The biofilms were then incubated for an additional 24 h. After the incubation of the bacteria with the compounds, the antibacterial activity of the compounds was assessed with a resazurin staining assay, followed by a crystal violet staining assay in the same plate as described in the following sections.

### 2.4. Resazurin Staining

For the compound library screening, resazurin staining was performed in 384-well plates according to the protocol previously optimized in our group [21] with following modifications. Briefly, the planktonic solution was first transferred into a new 384-well plate and the optical density (OD) of the planktonic solution was measured using a Varioskan LUX Multimode microplate reader (Thermo Scientific, Vantaa, Finland). Then, 2 µL of a 400 µM resazurin (R7017, Sigma-Aldrich) solution in phosphate-buffered saline (PBS, BR0014, Thermo Fisher Scientific) was added to the wells (for a final resazurin concentration of 20 µM). The plate was incubated in darkness for 3–5 min at room temperature with shaking (220 rpm) and fluorescence was measured at λ_excitation_ = 560 nm and λ_emission_ = 590 nm using top optics of the Varioskan LUX Multimode microplate reader. The biofilms, still in the original screening plates, were washed once with PBS to remove the remaining planktonic cells. Then, 40 µL of a resazurin solution at 20 µM (pre-exposure) or 40 µM (post-exposure) was added to the wells and the plates were incubated in darkness at room temperature (RT) for 40 min with shaking (220 rpm). Fluorescence was measured as previously described for the planktonic solution.

After the screening, subsequent resazurin staining experiments (dose–response and activity on additional strains) were performed in 96-well plates. The working volume in each well was of 200 µL (instead of 40 µL for 384-well plates) and the final resazurin solution concentration was 20 µM for all bacterial strains. For Gram-positive strains, an incubation of 30 min at RT was sufficient for the biofilms. For Gram-negative strains, the biofilms were incubated at 37 °C for a longer time ranging from 1 to 1.5 h. For all resazurin (and subsequent crystal violet) assays, untreated bacteria (negative control) and bacteria exposed to the solvent alone (DMSO 1–2.5%) were used as controls.

### 2.5. Crystal Violet Staining

The biofilms (previously stained with resazurin) were subjected to crystal violet staining as follows. The resazurin solution was removed and replaced by ethanol at 100% for 15 min at RT. The ethanol was removed, and biofilms were left to air-dry completely at RT. Then, crystal violet, diluted by 1:100 in deionized water from the 2.3% commercial solution (HT90132, Sigma-Aldrich), was added in the wells (35 µL in 384-well plates and 190 µL in 96-well plates). After 5 min, the biofilms were washed twice in deionized water and left to air-dry for about 10 min. The dye was solubilized in ethanol 100% for 1 h and absorbance was measured at 595 nm using a Multiskan Sky microplate spectrophotometer (Thermo Scientific, Vantaa, Finland). Crystal violet staining experiments subsequent to the screening (dose–response and activity on different strains) were performed in 96-well plates at a working volume of 200 µL per well.

### 2.6. Minimum Bactericidal Concentration (MBC) and Biofilm Preventing Concentration (BPC)

Minimum bactericidal concentration (MBC) is described here as the lowest concentration killing >99% of planktonic bacterial cells (one or no colony after overnight incubation on agar) and biofilm preventing concentration (BPC) as the lowest concentration to prevent the adherence and survival of >99% of bacterial cells on the surface of the wells. *S. aureus* was grown in a 96-well plate and exposed to fingolimod (same concentrations as for the dose–response for each strain) in pre-exposure as described in the culture conditions section. After 18 h, in wells where no visible growth was observed (from the MIC and higher), a volume of 50 µL of planktonic solution was transferred directly on a TSA plate (MBC determination). The wells were then emptied of the remaining planktonic solution and washed once with PBS. The bottom of the wells was scraped in 50 µL of PBS using a pipette tip and the full volume was transferred onto a TSA plate (BPC determination). All TSA plates were incubated overnight at 37 °C and the presence of colonies was assessed on the next day. Untreated bacteria (negative control) and bacteria exposed to the solvent alone (DMSO 1%) were used as controls.

### 2.7. Quantification of the Viable Cells in Biofilms

To quantify the activity of fingolimod on pre-formed biofilms (post-exposure) in terms of viable cells remaining after treatment, biofilms were grown in 96-well plates in 200 µL of TSB for 18 h at 37 °C with shaking and then exposed to the compound for 24 h in the same incubation conditions. The planktonic solution was removed from the wells and biofilms were washed once with PBS before being scraped in 100 µL of PBS with a pipette tip. Then, the bacteria were serially diluted in PBS and plated on TSA in 10 µL drops. The washing of the biofilms with PBS and serial dilutions reduced compound carryover onto the TSA plates. CFUs were counted after an incubation at 37 °C overnight. Untreated bacteria (negative control) and bacteria exposed to the solvent alone (DMSO 1%) were used as controls.

### 2.8. Time-Kill Kinetic Assay

The bacterial culture was made as explained above, however, the cells were grown overnight instead of a few hours. On the next day, it was diluted to a concentration of 1 × 10^6^ CFU mL^−1^. In 15 mL Falcon tubes (62.554.502, Sarstedt, Nümbrecht, Germany), 40 µL of fingolimod at different concentrations (0.5 × MIC, MIC (12 µM), 2 × MIC, 4 × MIC, 16 × MIC) were added to 4 mL of bacterial culture. For the growth control, bacteria were exposed to the solvent alone (DMSO 2%). The tubes were then incubated at 37 °C with shaking (220 rpm) and a sample of 200 µL was collected from each tube into a 1.5 mL tube (72.690.550, Sarstedt) at various time points (15 min, 30 min, 1, 2, 3, 4, 6, 8, 12, and 24 h). The samples were centrifuged at 10,000× *g* for 5 min at 4 °C and the supernatant was discarded to limit compound carry-over. Bacteria pellets were resuspended in 200 µL of PBS and transferred into a 96-well plate for an OD measurement (595 nm) using the Varioskan LUX Multimode microplate reader. Bacteria were then serially diluted in PBS and 10 µL drops were seeded on TSA plates. After an overnight incubation at 37 °C, colonies were counted.

### 2.9. Assessing Resistance Development

The bacterial culture was made like described above but it was grown overnight instead of a few hours. On the next day, it was diluted to a concentration of 1 × 10^6^ CFU mL^−1^. In a 96-well plate, 200 µL per well of the bacterial culture were added in wells containing the compounds (fingolimod and three antibiotic controls; doxycycline, penicillin, oxacillin) at either the MIC or half of the MIC. The plate was incubated at 37 °C with shaking (220 rpm) for 24 h, after which the bacterial growth was visually assessed. If bacterial growth was visible in the highest concentration of compound, that would indicate an increase in the MIC (resistance development) and 10 µL of that bacterial culture was transferred into two new wells containing 190 µL of TSB with either the same concentration or the double concentration of compound. If bacteria only grew in the well containing 0.5 × MIC, the compound concentrations in the new wells were kept the same. The plate was then incubated again for 24 h and the process was repeated for 20 days.

### 2.10. Quorum Sensing Inhibition Assays

The ability of fingolimod to block the QS system in Gram-negative bacteria was assessed essentially as described previously [23,24]. Shortly, two QS reporter strains, *C. violaceum* ATCC 31532 and the violacein-negative, mini-Tn5 mutant of *C. violaceum* CV026 were grown on LBA (for CV026 the agar was supplemented with kanamycin at 100 µg mL^−1^) overnight at 27 °C. Colonies from LBA were suspended in PDYT (0.5% peptone, 0.3% d-glucose, 0.25% yeast extract, 0.05% l-tryptophan, *w/v*) to achieve OD_600_ = 0.02. In the case of CV026, the cell culture was supplemented with C6-HSL (*N*-hexanoyl-l-homoserine lactone) (10007896, Cayman Chemical, MI, USA) at 0.5 µM to induce the QS system and subsequent synthesis of violacein, the indicator of QS induction. Then, 200 µL of bacteria culture was added per well in two identical 96-well plates containing the compounds (2 µL per well for a final DMSO concentration of 1% as the cell control showed unaffected violacein production). In each 96-well plate, the untreated cells were used as negative controls and quercetin (Q4951, Sigma-Aldrich) and azithromycin (PZ0007, Sigma-Aldrich) were included as positive controls for QS inhibition and cell viability (bactericidal activity), respectively. The plates were incubated at 27 °C under aerobic conditions (200 rpm) for 22 h.

The synthesized and non-soluble violacein was collected from the first 96-well plate by centrifugation (4000 rpm, for 20 min, 20 °C) and the supernatants were removed. The violacein was dissolved in 96% (*v*/*v*) ethanol and separated from cells by centrifugation as described above. Aliquots of 100 µL containing the violacein were transferred to a new 96-well plate and changes in violacein production were monitored at λ = 595 nm using a Multiskan Sky Microplate spectrophotometer. Bactericidal effects were detected using the second replica plate by adding 10 µL of 400 µM resazurin in each well and incubating the plate for 30 min in the darkness at 27 °C (220 rpm). After centrifugation, 100 µL of each well was transferred into a new 96-well plate and the changes in viability (i.e., reduction of resazurin into resorufin) was recorded as described above using the Varioskan LUX multimode plate reader.

### 2.11. Statistical Analysis

Screening performance was monitored by the calculation of the screening window coefficient (Z’) as described in [25]. The statistical significance of the results was assessed using a Student’s *t*-test and significance was indicated as follows: * if *p* < 0.05, ** if *p* < 0.01 and *** if *p* < 0.001.

## 3. Results and Discussion

### 3.1. Screening FDA-Approved Compounds Identifies 45 Anti-Biofilm Agents against S. aureus

The anti-bacterial screening of the FDA library was performed in 384-well plates. The ability of the compounds to prevent bacterial growth and biofilm formation (pre-exposure) as well as affect pre-formed biofilms (post-exposure) was separately measured at 10 µM. The identities of the tested compounds and their activities during the primary screenings are listed in the Supplementary Table S1.

**Figure 1:**
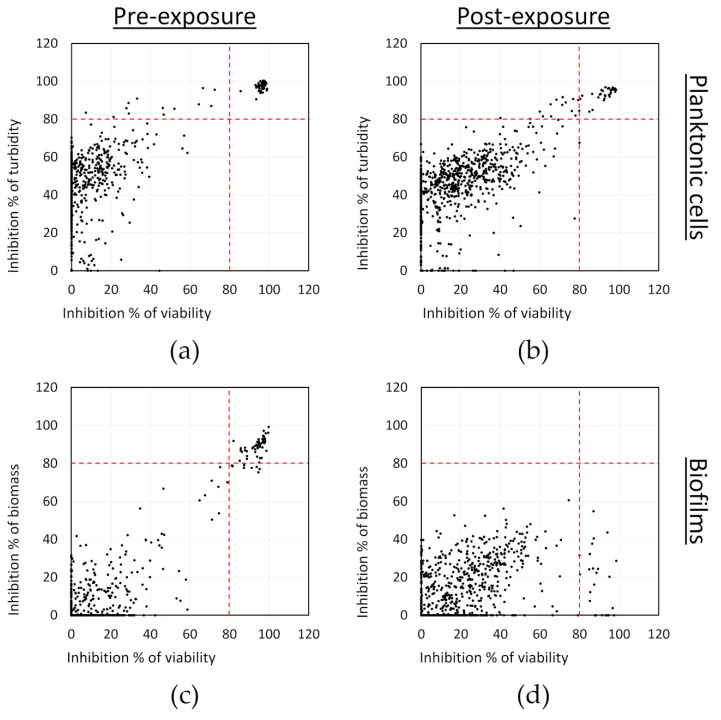
Screening of an FDA-approved compound library against *S. aureus* in pre-exposure (**a** and **c** panels) and in post-exposure (**b** and **d** panels): (**a**,**b**) inhibition of planktonic cell viability and turbidity; and (**c**,**d**) inhibition of the biofilm biomass and viability. Results are averages from two separate experiments with each one replicate per compound.

The effect of the compounds on planktonic cells was monitored by measuring the turbidity of the cells (OD at 595 nm) and the viability by resazurin staining. In the case of the biofilms, we also used resazurin staining for monitoring changes in viability, while crystal violet staining was applied to measure changes in total biomass. The effects of the tested compounds on both the planktonic cells and biofilms in comparison to untreated bacterial controls are shown in Figure 1. A threshold of 80% inhibition was empirically set to select the active compounds. When considering the turbidity and viability results, 59 compounds were identified as potential hits against the planktonic cells in the pre-exposure mode of assay. A lower number of compounds (45) were identified as potential anti-biofilm hits, which reduced both the viability and biomass production of *S. aureus* by at least 80% in the pre-exposure mode of assay. These 45 compounds were also among the hits against planktonic cells, except for one that was active in preventing biofilms but not against planktonic cells, for a total of 60 hits in the pre-exposure assay. In the post-exposure assay, no new compounds were identified with specific effects on already pre-formed biofilms. Overall, 32 compounds were active against planktonic cells in post-exposure, all of which were also identified as hits in the pre-exposure assay. From these, 24 were found to reduce more than 80% of the viability of the pre-formed biofilms, but none of these compounds considerably reduced the total biomass of the pre-formed biofilms. This is not unexpected as mature biofilms, as mentioned earlier, are known to be very tolerant to antimicrobial chemotherapy.

### 3.2. Hit-to-Lead Identification Process

**Figure 2:**
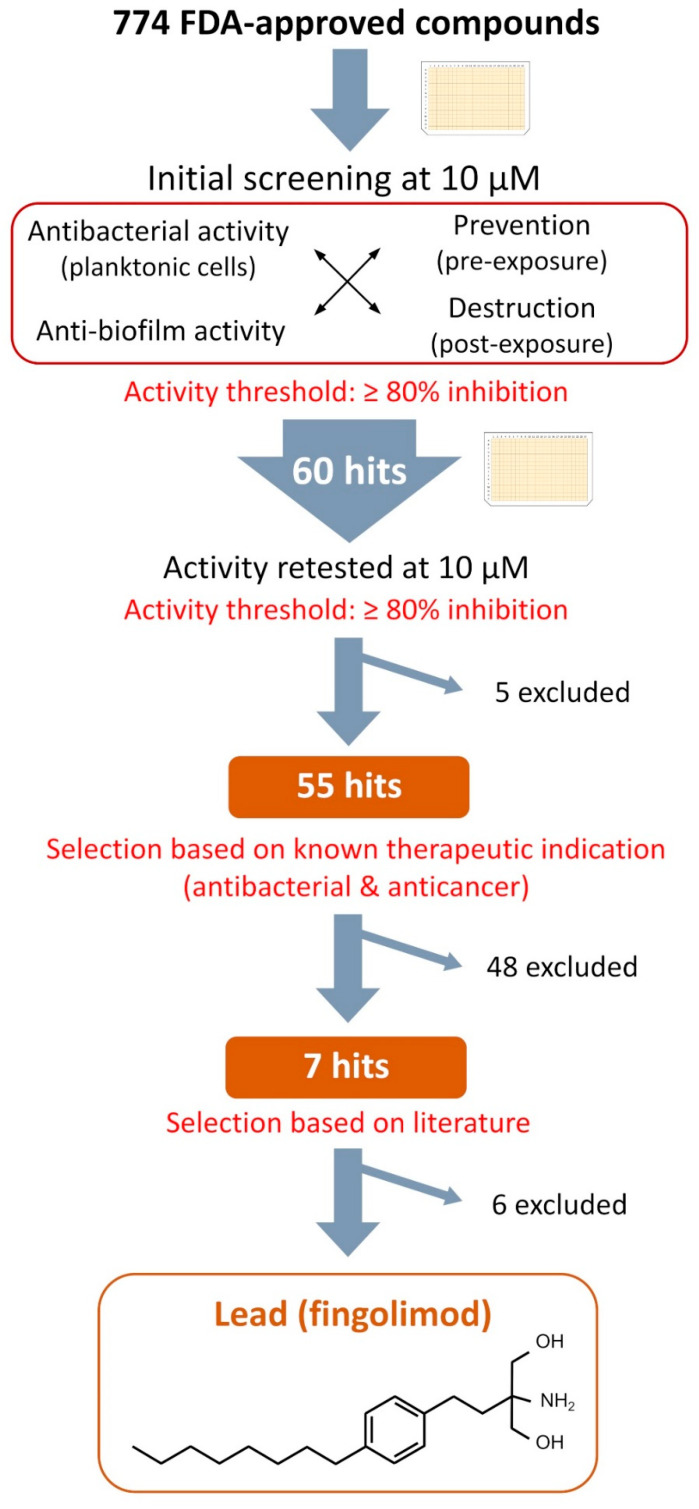
Selection process from the initial screening of the compound library to the selection of the lead and chemical structure of fingolimod.

Figure 2 summarizes the selection process carried out after the primary screening. As described in the previous section, a total of 60 hit compounds were identified in the initial screening against either the planktonic cells (turbidity and viability), the biofilms (viability and biomass production) or both. First, these 60 hits were retested at 10 µM in a 384-well plate (Supplementary Table S2) with the same combination of assays used during the primary screening to confirm the screening results. Following this, five initial hits were readily excluded as they did not show an inhibition of the planktonic cells nor the *S. aureus* biofilms over 80%. The known therapeutic indications of the remaining 55 confirmed hits were explored. Based upon this, it was learned that 48 of these hits were listed in the information provided by the library supplier as being either approved antibiotics or already widely known for their antibacterial activity (41), or they had been approved for the treatment of cancer (7) and were excluded due to their likeliness to have high cytotoxicity.

For the seven remaining hits, a literature search was undertaken to evaluate their potential as repurposed antibacterial agents. We identified four active hits (sertaconazole, econazole, miconazole and sulconazole), which were approved antifungal drugs of the imidazole class. They are known to inhibit the synthesis of ergosterol, the main sterol of fungal membranes, as well as other important components [26,27]. Imidazoles are currently the focus of numerous medicinal chemistry efforts and many derivatives have shown potent antibacterial and anti-biofilm activity [28,29,30,31]. Therefore, it is not surprising that they were also identified as positive hits in our study. Of the remaining three hits, one was auranofin, a gold-containing drug, which has been approved for the treatment of rheumatoid arthritis. Auranofin’s potential as a repurposed antimicrobial agent against various organisms, including *S. aureus*, has already been explored [32,33], and it was therefore excluded from further analysis. The second remaining hit was acyclovir, a known antiviral commonly used for the treatment of viruses of the herpes family, which to the best of our knowledge has not yet been reported as an anti-bacterial compound, and is currently being studied in detail by our group.

The last identified hit compound was fingolimod, and it was selected as the lead for further characterization. Fingolimod (Figure 2), 2-amino-2-[2-(4-octylphenyl)ethyl] propane-1,3-diol (FTY720) is an analogue of sphingosine 1-phosphate (S1P) that has been approved for the treatment of relapsing multiple sclerosis. It is a synthetic derivative of a natural compound, myriocin, isolated from the fungus *Isaria sinclairii* [34,35]. It acts as an antagonist to S1P receptors located on lymphocytes and prevents those cells leaving the lymph nods, causing a modulation in the immune response through a redistribution of the lymphocytes [36]. On the other hand, sphingosine is a common sphingoid base naturally occurring in mammalian cells. The sphingoid base is the structural unit of sphingolipids, which are structurally diverse molecules involved in many biological functions such as inflammation, cell differentiation and apoptosis [37,38]. The therapeutic potential of sphingoid bases and sphingolipids has been widely explored for the treatment of a variety of diseases, including cancer, ischemia and immune diseases [39,40,41]. Interestingly, sphingosine has been found to be important in the immune defense of healthy airways as a lack of sphingosine increased their susceptibility to bacterial infections, while the administration of sphingosine has been shown to prevent or reduce bacterial infection [42,43,44,45]. Sphingosine has also been reported to act as an antibacterial amino alcohol in the skin [46,47,48]. Overall, many recent studies have proved that sphingosine and some of its derivatives have antibacterial activity against a variety of species including, among others, *P. aeruginosa*, *Acinetobacter baumannii*, *Escherichia coli*, *S. aureus* and *Streptococci* species [42,44,46,47,49,50,51]. A sphingosine coating of endotracheal tubes has even recently been tested against *P. aeruginosa*, *A. baumannii* and *S. aureus* and it showed a great capacity to prevent bacteria adherence and protection from ventilator-associated pneumonia (VAP) in vivo [52]. A recent MoA study has reported that sphingosine causes the rapid permeabilization of the bacterial membrane of *P. aeruginosa* and *S. aureus* through the binding of sphingosine’s protonated nitrogen group with cardiolipin, a negatively charged membrane protein [53]. In particular, fingolimod’s therapeutic potential has been investigated for many conditions, mainly for various autoimmune diseases [54,55]. However, its antibacterial activity has not been extensively studied, although this drug is reported to display a potent activity against planktonic cells of the gut bacteria *Clostridium perfringens* and to provide in vivo protection against *P. aeruginosa* [43,56]. Even though conclusions from studies made on various fingolimod structural analogues could probably be applied to fingolimod, the activity of fingolimod itself against Gram-positive strains and biofilms has not been studied, to the best our knowledge, and little is known of its MoA. Based upon this, we set out to investigate the extent of fingolimod’s antibacterial activity and to gather information on its mechanism of action.

### 3.3. Concentration–Response Effect of Fingolimod against S. aureus Biofilms

**Figure 3:**
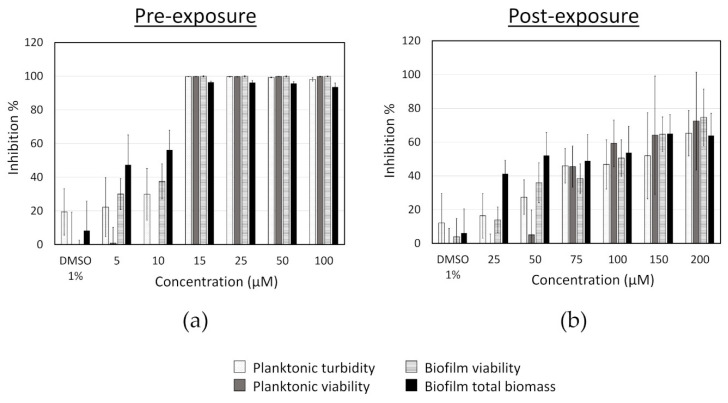
Inhibition of *S. aureus* in pre-exposure (**a**) and in post-exposure (**b**) assays caused by different concentrations of fingolimod. Results are expressed as the average inhibition percentage of the planktonic cells or the biofilms in comparison with the untreated controls ± SD. Results are from four distinct experiments with two to three technical replicates each.

First, the effect of different concentrations of fingolimod on *S. aureus* biofilms was investigated using the pre- and post-exposure assay modes with resazurin and crystal violet assays (Figure 3). The MIC of fingolimod (lowest concentration at which no growth was visible and at which the OD of the planktonic cells was the same as the media control (inhibition of 100%)) was measured to be between 12.5 and 15 µM (depending on the replicates), at which concentration fingolimod prevents both the growth of planktonic cells and biofilm formation. In post-exposure, although not completely disrupting the biofilms, fingolimod at 200 µM did have a substantial effect on the pre-formed 18 h-old biofilms, as it reduced the viability and total biomass by about 70–80% after 24 h of incubation.

We also verified if the pre-exposure activity of fingolimod was bacteriostatic or bactericidal. *S. aureus* was exposed to fingolimod for 18 h in the same way as for the concentration–response experiment. In wells where no bacterial growth was visible (exposed to a fingolimod concentration above the MIC), the planktonic as well as possible adherent cells scraped from the bottom of the wells were separately transferred onto TSA plates for measuring viable cells (CFU counts) after an overnight incubation (Table 2). This allowed confirming that fingolimod does kill all *S. aureus* cells and almost completely prevents the formation of adherent colonies at a concentration as low as 15 µM, which is therefore not only the MIC, but also the minimum bactericidal concentration (MBC) and the biofilm preventing concentration (BPC). MBC is defined here as the lowest concentration killing >99% of planktonic bacterial cells (one or less colony after overnight incubation on agar) and BPC as the lowest concentration to prevent the adherence and survival of >99% of bacterial cells on the surface of the wells.

### 3.4. Reduction in the Count of Viable Cells in Pre-Formed S. aureus Biofilms Exposed to Fingolimod

We utilized resazurin and crystal violet assays to measure the effects of fingolimod on the viability and biomass of *S. aureus* biofilms. Crystal violet was performed on fixated biofilms and dyes, all negatively charged surfaces, including the dead cells and the remaining matrix. In contrast, the redox-based resazurin staining does discriminate live cells from dead cells via the specific reduction of resazurin into its fluorescent product resorufin by live cells. As this assay depends on the metabolically active cells, the dormant cells with reduced/lacking metabolic activity fail to be detected. Because biofilms are communities composed of cells displaying various degrees of metabolic activity, including nearly inactive/dormant cells, this means that an important part of a biofilm’s population cannot be typically detected with resazurin. Among those cells are also persisters, which are unaffected by most antibacterial compounds and can reactivate and restart growth after antimicrobial treatment [57]. Furthermore, it is possible that resazurin does not penetrate all biofilms with the same kinetics, resulting in a lower “viability” being detected at a certain time point, despite the potential presence of live cells deeper inside the biofilms. Therefore, even if resazurin staining indicates that a biofilm is entirely inactive, the possibility that some cells are still alive cannot be precluded.

**Figure 4:**
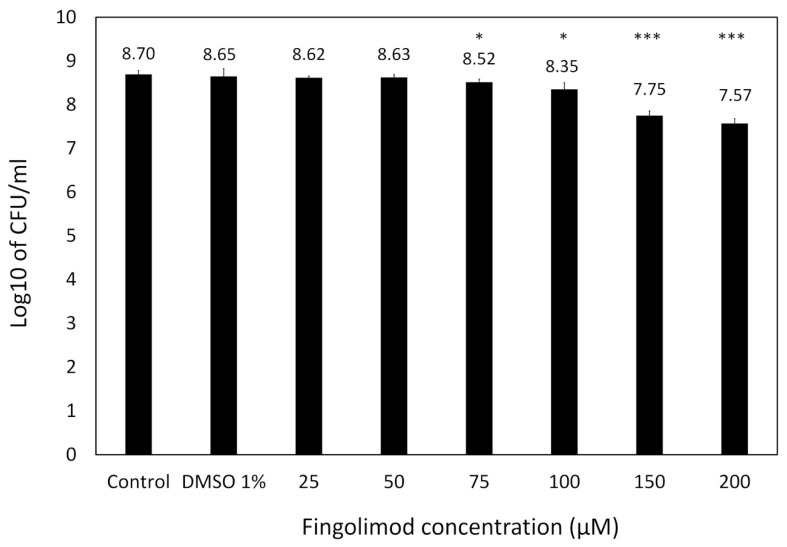
Log10 of the CFU count of *S. aureus* pre-formed biofilms treated with different concentrations of fingolimod (post-exposure) for 24 h. Untreated biofilms are used as controls. Results are from three distinct experiments, each with two to three biofilm replicates for all concentrations ± SD. (* *p* ≤ 0.05, *** *p* ≤ 0.001).

Based upon this, we decided to further quantify the activity of fingolimod on pre-formed 18 h-old *S. aureus* biofilms by performing a viable cell count after a 24 h-fingolimod treatment. This would allow us to confirm if the 70–80% inhibition previously observed (Figure 3) would correspond to a similar level of reduction in the viable cell count. Figure 4 shows that fingolimod reduces the number of viable cells inside a pre-formed biofilm in a concentration-dependent manner down to a statistically significant 1-log reduction at 150–200 µM, which represents a 90% reduction in the number of cells. This confirms that fingolimod does reduce the count of live cells within a pre-formed biofilm and it is aligned with the previous results of the resazurin and crystal violet staining.

### 3.5. Time-Kill Kinetic Effect of Fingolimod against S. aureus

**Figure 5:**
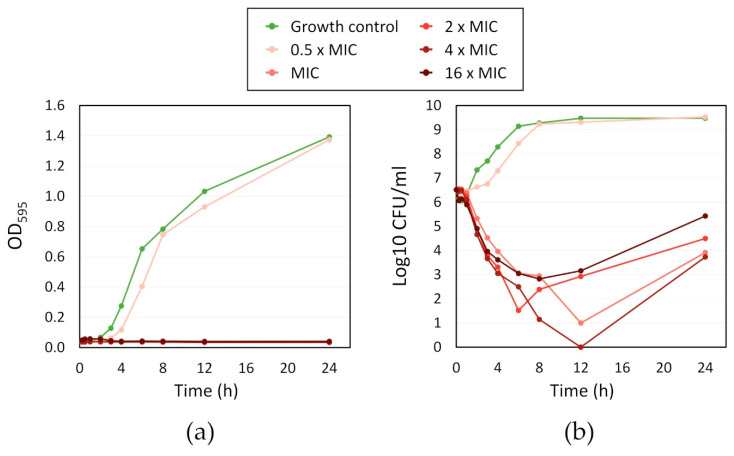
Time-kill kinetic of different folds of the minimum inhibitory concentration (MIC) (12 µM) of fingolimod against *S. aureus*: (**a**) OD595 of the bacterial culture over time; and (**b**) Log10 of the CFU/mL in the bacterial culture over time. Results are the average of two distinct experiments with each one replicate per concentration.

For a more in-depth investigation of the activity of fingolimod against *S. aureus*, we performed a kinetic (time-kill) study by counting the CFU and measuring the OD of the bacterial culture over time when exposed to different concentrations of fingolimod (ranging from 0.5 × MIC to 16 × MIC) with MIC set at 12 µM for this experiment (Figure 5). The changes in OD over time demonstrate that no concentration of fingolimod from the MIC and higher allowed visible growth of the bacteria for 24 h and only the culture containing 0.5 × MIC (6 µM) had an increasing OD similar to the growth control. The other tested concentrations of fingolimod had a similar effect on the *S. aureus* culture, with a rapid decrease in the number of live cells starting within 2 h of incubation. All concentrations from the MIC and above caused at least a 3-log reduction or more, after 12 h. However, the amount of CFU always started increasing between 12 and 24 h, suggesting that, even when no CFU were counted at certain time points, a small fraction of cells survived the treatment and started to grow again after the treatment started to lose efficacy. The survival of those cells with all fingolimod concentrations is not unusual as it has been proven that within all populations of bacteria, there are always persisters that survive treatment, regardless of the concentration of antibiotic used [57]. Another explanation could be the rapid development of resistance against fingolimod by some of the cells in the cultures, a possibility that we investigate here, further on. This time-kill experiment demonstrates the strong ability of fingolimod to reduce the number of bacteria in a culture in a short period of time. Our findings show that fingolimod becomes active during the early exponential growth phase (after 2 h of incubation), indicating that its MoA is linked with the inhibition of one or more key metabolic pathways.

### 3.6. Resistance Development of S. aureus against Fingolimod

With the development of antibacterial resistance, the so-called “super bugs” have emerged, which are resistant to most, if not all, available antibiotics [3]. In this context, and considering the results obtained in the time-kill assay, we then decided to investigate whether *S. aureus* develops resistance against fingolimod by exposing the cells to a sub-inhibitory concentration of the drug in a 20 day period. Well characterized model antibiotics (doxycycline, penicillin G and oxacillin) were used as references and the bacteria were exposed to the MIC and 0.5 × MIC every day. After 24 h, if bacterial growth was visible, some bacteria were transferred into new media containing the same concentration as well as a 2-fold concentration of the compound. This was repeated for 20 days to see if the MIC would increase over time.

**Figure 6:**
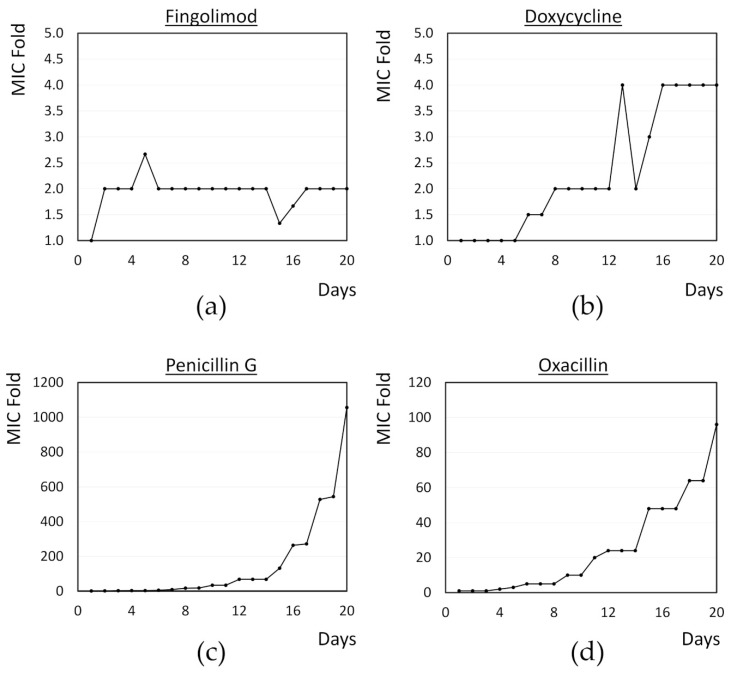
MIC doubling over time for *S. aureus* exposed to sub-inhibitory concentrations of (**a**) fingolimod; (**b**) doxycycline; (**c**) penicillin G; and (**d**) oxacillin for 20 days. Results are the average obtained for two to three biological replicates.

Results of the resistance development are shown in Figure 6. As earlier, the MIC of fingolimod was set at 12 µM and it quickly increased by 2-fold by day 2. However, no further increase in the MIC was observed for the whole duration of the experiment. In contrast, a 4-fold increase in the MIC was observed for doxycycline, the best performing control in this study, while an increase of nearly 100-fold and 1000-fold was observed for oxacillin and penicillin G, respectively. Considering that the MIC of fingolimod can slightly vary between 12 and 15 µM in each experiment, it is possible that the 2-fold increase in the MIC observed here was not an indication of resistance development, but simply of the MIC being set slightly too low at the beginning of the study. The absence of any further increase in the MIC after that early doubling support this possibility. Overall, this indicates that resistance would only be developing slowly against fingolimod and that its mechanism or its interaction with the target is likely to be different from those of the control antibiotics. This finding further advocates that fingolimod could also be used as an antibacterial drug, as it does not induce resistance and could even be used in combination with other antibiotics.

### 3.7. Activity of Fingolimod on Additional Bacterial Strains

**Figure 7:**
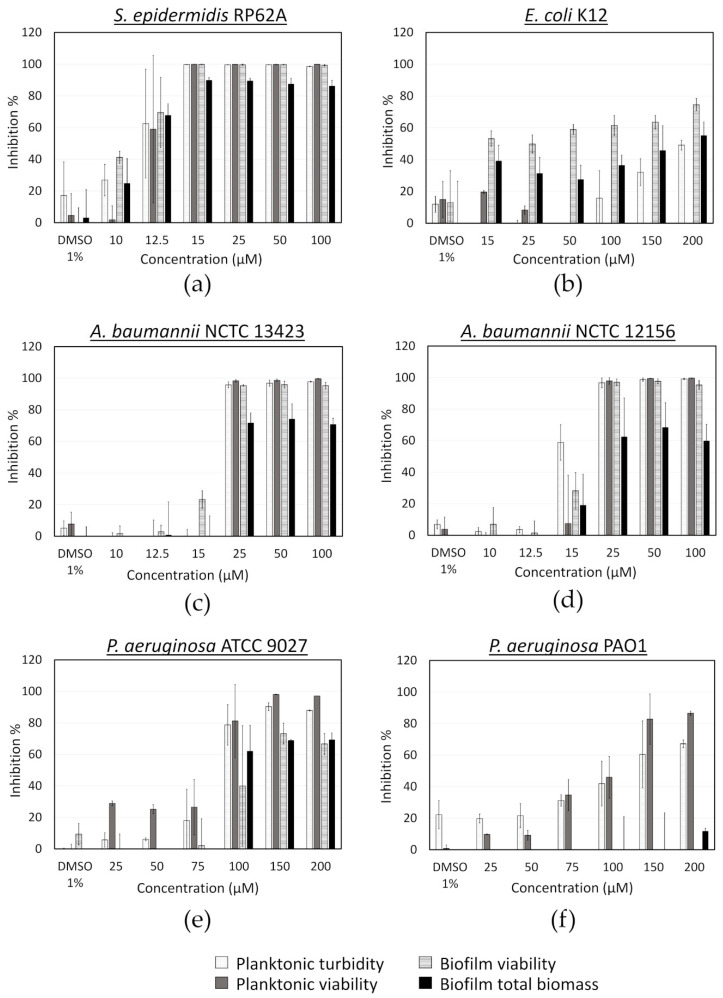
Activity of fingolimod against different bacterial species in the pre-exposure mode. Results are from two distinct experiments with each two or three biofilm replicates for all concentrations ± SD.

Further on, we investigated the spectrum of the antibacterial activity of fingolimod by testing it against different pathogenic bacterial species, such as Gram-positive *S. epidermidis* and Gram-negative *A. baumannii*, *E. coli* and *P. aeruginosa*. In the case of *A. baumannii*, we used two different strains for this experiment, the reference strain National Collection of Type Culture (NCTC) 12156 and the multi-drug resistant and strong biofilm producer NCTC 13423 [58]. Two different clinical *P. aeruginosa* strains, ATCC 9027, a non-virulent antibiotic-sensitive strain [59] and PAO1, a strain widely used in research as a model opportunistic pathogen, were tested in addition to two environmental strains, ATCC 700829 and ATCC 15442. The activity of fingolimod on *S. epidermidis* was very similar to what was observed with *S. aureus,* as evidenced with the resazurin and crystal violet assays (Figure 7). The compound was also quite effective against *A. baumannii*, with an MIC of 25 µM for both strains, indicating that fingolimod is not affected by the resistance mechanisms of the *A. baumannii* NCTC 13423 strain. The bactericidal activity of fingolimod against the multi-drug resistant *A. baumannii* NCTC 13423 strain, was further confirmed using viable cell counts, in a similar way as with *S. aureus* (Table 3). Fingolimod killed all planktonic cells (MBC) in 78% of the replicates between at 100 µM, but it did not prevent the survival of all adherent cells (BPC) at any concentration up to 100 µM.

**Figure A1:**
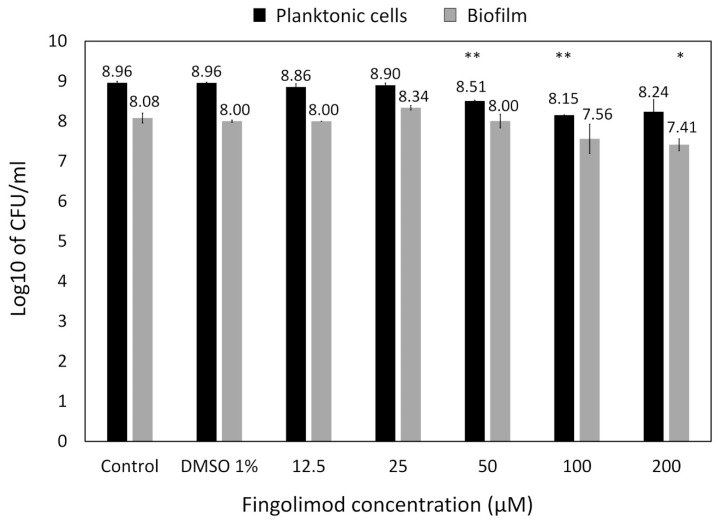
CFU count of *E. coli* planktonic solution and biofilm after an 18 h treatment with fingolimod in the pre-exposure mode. Results are from two distinct experiments with each three biofilm replicates for all concentrations ± SD.

A lower inhibitory activity was observed against *E. coli* (Figure 7), which was mainly targeted at biofilm formation, while no planktonic bacteria appeared to be killed. This could be due to fingolimod reducing biofilm formation, by QS inhibition for example, or affecting biofilm integrity, making it more brittle and resulting in some biofilm being broken and lost during manipulations. A viable cells (CFU) count was done for both planktonic cells and biofilms after treatment with fingolimod for 18 h to see if the inhibition of the *E. coli* biofilms was also translating into a reduction in the viable cells count. Fingolimod reduced by less than a log the amount of CFU from both the planktonic solution and the biofilms, thus, although some biofilm-specific inhibition is suggested by the resazurin and crystal violet assays, it does not translate into a large reduction in the amount of live cells within the biofilm (Figure A1).

Fingolimod also showed a limited activity against certain strains of *P. aeruginosa*. It visibly reduced the planktonic and biofilm growth of *P. aeruginosa* ATCC 9027 from 100 µM, but only inhibited the planktonic cells of PAO1 without affecting the biofilm formation (Figure 7). Since ATCC 9027 strain is known as antibiotic-sensitive, it is not surprising to find that this strain is the most susceptible to fingolimod, while the pathogenic PAO1 is more tolerant to it. In addition, fingolimod displayed no important inhibitory activity against the two environmental strains of *P. aeruginosa* (ATCC 700829 and ATCC 15442) (Supplementary Figure S1). Fingolimod has previously been reported to prevent *P. aeruginosa* airway infection in mice and to have a strong in vitro activity against this species with an EC_50_ of 1.9 µM [43]. We did not observe an activity nearly as strong, but a difference in the bacterial strains and the experimental conditions might explain this discrepancy. Overall, these results indicate that fingolimod has a broad spectrum of activity, with a stronger activity against Gram-positive than Gram-negative bacteria. Fingolimod displayed here a lower activity range than what has been observed for its analogue sphingosine and the susceptibility of the different species was also quite different than what was reported for sphingosine [49,53].

**Figure A2:**
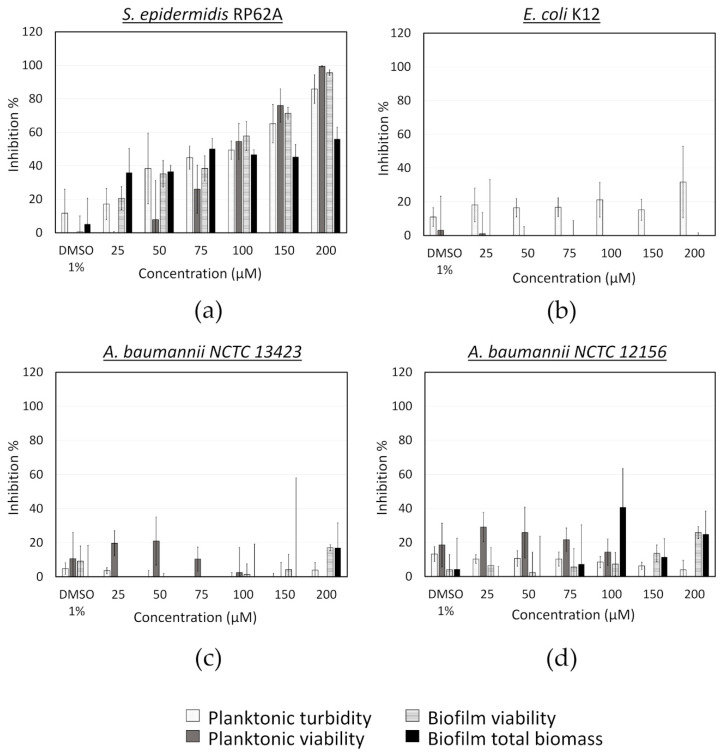
Activity of fingolimod against different bacterial species in the post-exposure mode. Results are from two distinct experiments with each two biofilm replicates for all concentrations ± SD.

Fingolimod was also tested against *S. epidermidis*, *A. baumannii* and *E. coli* using the post-exposure assay (Figure A2). In the case of *S. epidermidis*, the results were aligned with those obtained for *S. aureus*. However, in the case of the Gram-negative species, fingolimod did not show any activity, which is not surprising considering the well reported increase in resistance and tolerance factors expressed in mature bacterial communities [5,8,60]. It is possible that the penetration of fingolimod into the biofilm could be affected. Alternatively, if the MoA of this compound is based on affecting metabolic pathway(s), a lower metabolic activity within the biofilm could be responsible for the compound’s lower efficacy against a pre-formed biofilm [9].

### 3.8. Quorum Sensing Inhibitory Effect of Fingolimod

We then investigated the possible effect of fingolimod on QS, an important bacterial communication system that allows the coordination of many social behaviors, like the formation of a biofilm, when the population reaches a certain cellular density [61,62]. The molecules used for QS are called autoinducers (AIs) and in Gram-negative bacteria, they are generally acyl-homoserine lactones (AHLs), while Gram-positive bacteria use small peptides and both types of bacteria can use another kind of molecule called AI-2 [61,62]. *C. violaceum* is a Gram-negative strain that serves as an excellent model for the study of QS inhibitors (QSIs), since one of its QS-regulated behaviors is the production of a violet pigment called violacein. Its AHL signal is based on CviI/CviR, an homolog of the LuxI/LuxR synthase and receptor system that was first described in Gram-negative bacteria [63]. Rather than only recognizing the AHL from its corresponding synthase CviI, CviR has been reported to respond to a wider range of AHLs that have side chains from four to eight carbons in length [63,64].

Fingolimod’s chemical structure (Figure 2) resembles some of the previously reported antagonists of the receptors in the LuxR family and one of the AHL analogs able to inhibit AHL synthesis by binding to its synthase [64,65]. This similarity in structure with known inhibitors of QS suggests that fingolimod could be a QSI and although we observed that fingolimod does have a bactericidal activity against certain Gram-negative strains like *A. baumannii*, a biofilm-specific inhibition was observed against *E. coli*. Based upon this, we decided to investigate the possibility that fingolimod might be a QSI for some Gram-negative strains.

A platform based on the use of C. *violaceum* as a reporter strain was previously optimized and has been successfully used in chemical screens by our group [23]. This platform incorporates the measurement of both cell metabolic activity and violacein production in *C. violaceum* ATCC 31532, a wild-type strain, as well as CV026, a mutant that is AHL-deficient and needs an external AHL (C6-HSL) to be supplemented in order to produce violacein. The addition of the metabolic activity measurement allows to confirm that the QS inhibition observed is not due to toxicity and the use of CV026 allows the distinction between QS inhibitors (QSIs) and quenchers of the AHL signal, called quorum-quenchers (QQ) [23,66]. While bactericidal compounds will inhibit the metabolic activity of the cells, QSIs (including QQs) will only inhibit the violacein production.

**Figure 8:**
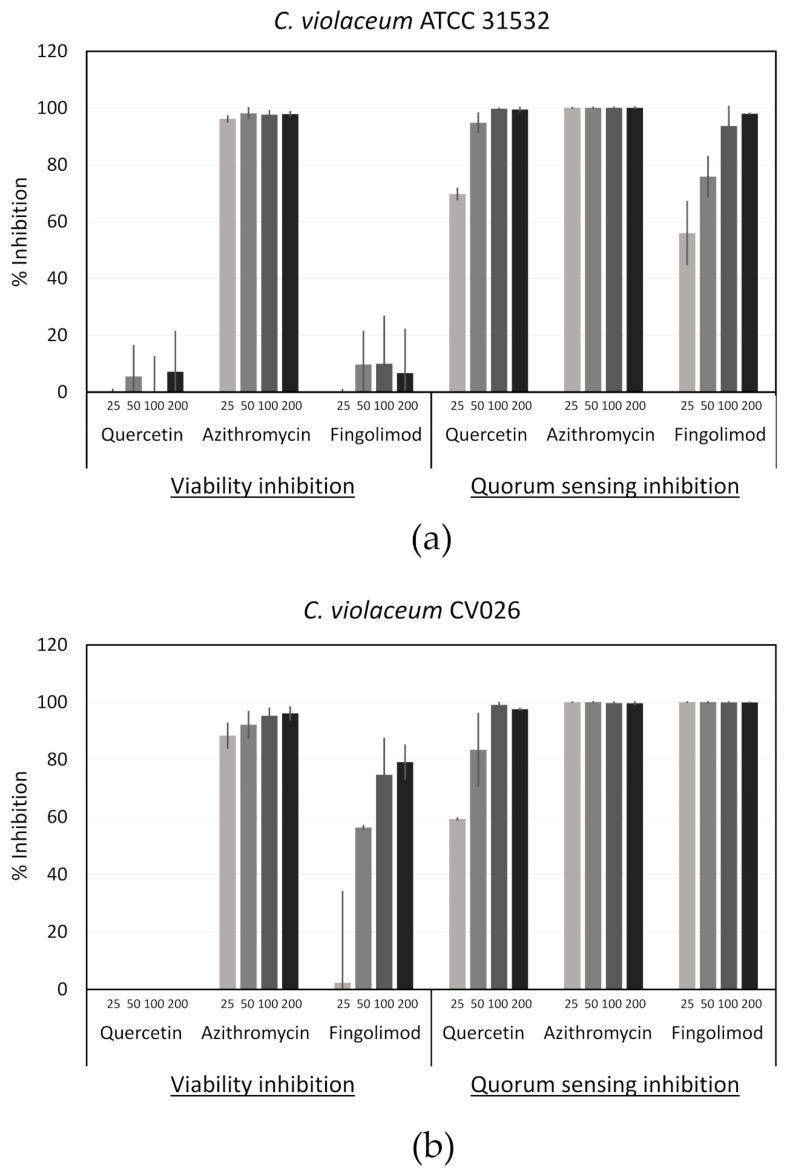
Viability and quorum sensing (QS) inhibition of *C. violaceum* (**a**) ATCC 31532 and (**b**) CV026 by fingolimod, quercetin (QS inhibitor (QSI) control) and azithromycin (bactericidal control). Concentrations are in µM and the results are expressed as percentages of inhibition in comparison with untreated controls. Results are from two different experiments performed with two technical replicates for each condition ± SD.

Using this platform, we tested fingolimod at various concentrations (Figure 8), along with the QSI control quercetin, a flavonol with a reported QSI activity, and a bactericidal control (with no QSI activity), azithromycin [67]. Fingolimod showed no noteworthy reduction in the metabolic activity of the wild type strain, but it did show a clear concentration-dependent inhibition of the violacein production. This demonstrates that fingolimod acts as a QSI against *C. violaceum*. However, while the violacein production of the mutant strain CV026 was completely inhibited from 25 µM onward, the metabolic activity of that strain was also decreased in a concentration-dependent manner. Therefore, especially with high concentrations, in addition to being a QSI, fingolimod seems to be also bactericidal against this strain. Nevertheless, the QSI effect was much stronger than the reduction in viability at 25 µM, which suggests that, at this concentration and below, fingolimod targets the QS in the reporter strain. The different results obtained with both strains of *C. violaceum* might reflect a different susceptibility to fingolimod that is unrelated to the AHL-deficiency of CV026.

As the QS systems between Gram-positive and Gram-negative bacteria are different, it is highly likely that fingolimod shows no QS inhibition activity against Gram-positive bacteria. Moreover, QS molecules can vary within the Gram-negative species, suggesting that fingolimod may not act as a QSI against all Gram-negative bacteria [61,62]. On the other hand, the LuxI/LuxR homologs have been reported in more than 100 Gram-negative species and more than 200 species use AHLs as QS signals, which implies that fingolimod still has a broad QSI activity against Gram-negative bacteria [68]. Since QS control biofilm formation as well as the expression of many virulence and pathogenic factors, QSIs have been the subject of a lot of attention in recent years as a promising alternative strategy to fight biofilms. QSIs can keep bacteria in the more susceptible planktonic state, which can increase the efficacy of antibiotics in a combination therapy [69]. Additionally, since they do not target an essential function of the bacterium, they do not apply any direct selective pressure, limiting the risks of resistance development [68,69].

## 4. Conclusions

To find new repurposed antibacterial compounds, we screened an FDA-approved compound library containing 774 compounds against *S. aureus*. The activity on both the planktonic cells and biofilms was assessed to select hits that would be effective enough to prevent biofilm formation. From the total of 60 originally identified hits, we performed a selection process that led to the identification of fingolimod. Fingolimod is an analogue of sphingosine 1-phosphate (S1P) that is currently used in the treatment of relapsing multiple sclerosis. Its activity against bacterial biofilms has not been explored previously, and we showed here that it inhibits the growth of both the planktonic cells as well as the formation of *S. aureus* biofilms. We also provided proof of its potent bactericidal activity against staphylococcal strains and modest to strong activity against diverse Gram-negative species, including the multi-drug resistant *A. baumannii* NCTC 13423 strain. We showed that, fingolimod is likely to target metabolically active cells in Gram-positive strains as it starts showing the observable killing of viable cells during the early stages of growth. In Gram-negative strains, while fingolimod displayed a bactericidal activity against some strains, we present here the first report of fingolimod (or any sphingosine analogue) having a QS-inhibitory activity against *C. violaceum*. Additionally, some multi-drug resistant strains were susceptible to fingolimod, suggesting a different MoA than common antibiotics. Interestingly, no antibacterial resistance was developed by *S. aureus* against fingolimod during a 20 day study. Fingolimod has been proven safe and stable for use in humans, as it is already an approved drug. It can serve as a good starting structure for optimization as the existence of fingolimod analogues with higher antibacterial activity suggests. We provide solid in vitro data in support of exploring this family of compounds for antibacterial and anti-biofilm therapeutics.

## Figures and Tables

**Table 1:** Bacterial strains, providers and analysis performed with each strain.

Bacterial Strains	Provider	Analyses
*Staphylococcus aureus* ATCC 25923	Faculty of Pharmacy of the University of Helsinki, Helsinki, Finland	Primary screening and concentration–response follow-up, Minimum Bactericidal Concentration (MBC) and Biofilm Preventing Concentration (BPC) determination, time-kill kinetic, resistance development
*Staphylococcus epidermidis* ATCC 35984 (RP62A)		Concentration–response
*Escherichia coli* K12	HAMBI collection, Faculty of Agriculture and Forestry, University of Helsinki, Helsinki, Finland	Concentration–response, post-exposure CFU count
*Acinetobacter baumannii* NCTC 13423	National Collection of Type Culture (NCTC), Salisbury, United Kingdom	Concentration–response, MBC and BPC determination
*Acinetobacter baumannii* NCTC 12156		Concentration–response
*Pseudomonas aeruginosa* ATCC 9027	American Type Culture Collection (ATCC), Wesel, Germany	
*Pseudomonas aeruginosa* ATCC 700829		
*Pseudomonas aeruginosa* ATCC 15442		
*Pseudomonas aeruginosa* PAO1	Laboratory of Microbiology, Parasitology and Hygiene (LMPH), University of Antwerp, Antwerp, Belgium	
*Chromobacterium violaceum* ATCC 31532	ATCC, Wesel, Germany	Quorum sensing inhibition
*Chromobacterium violaceum* NCTC 13278 (Tn5-mutant CV026)	NCTC, Salisbury, United Kingdom	

**Table 2:** MBC and BPC confirmation of fingolimod against *S. aureus* after a pre-exposure treatment. Results are expressed as the percentage of replicates for which >99% of cells were killed (one or less colony counted) (*n* = 9, three separate assays with each three replicates).

% of Replicates with >99% Cells Killed		
(Fingolimod)	Planktonic Cells	Adherent Cells
10 µM	0	0
12.5 µM	11	11
15 µM	100 (MBC)	100 (BPC)
25 µM	100	89 ^1^
50 µM	100	89

^1^ One replicate out of nine had <99% cells killed.

**Table 3:** MBC and BPC confirmation of fingolimod against *A. baumannii* NCTC 13423 after a pre-exposure treatment. Results are expressed as the percentage of replicates for which >99% of cells were killed (one or less colony counted) (*n* = 9, three separate assays with each three replicates).

% of Replicates with >99% Cells Killed		
(Fingolimod)	Planktonic Cells	Adherent Cells
12.5 µM	0	0
25 µM	44	11
50 µM	56	11
100 µM	78	11

## Supplementary Materials

The following are available online at https://www.mdpi.com/2076-2607/8/11/1834/s1, Table S1: Compounds and results of the screening of the Screen-Well FDA Approved Drug Library Version 2 (ENZO Life Sciences) against *Staphylococcus aureus* ATCC 25923. Table S2: Compounds and results of the activity confirmation of the initial pre-exposure hits from the Screen-Well FDA Approved Drug Library Version 2 (ENZO Life Sciences) at 10 μM against *Staphylococcus aureus* ATCC 25923. Figure S1: Inhibition of Pseudomonas aeruginosa ATCC 15442 and 700829 in pre-exposure by different concentrations of fingolimod.
