# Supplementary material for: Screening of FDA-Approved Drugs Using a 384-Well Plate-Based Biofilm Platform: The Case of Fingolimod
Source: Microorganisms. 2020 Nov 21;8(11):1834. doi: 10.3390/microorganisms8111834 (PMC7700524; doi:10.3390/microorganisms8111834)
Supplement: Supplementary file 1 [file microorganisms-08-01834-s001.zip › Table S1.pdf]

Supplementary table 1. Compounds and results of the screening of the Screen-Well FDA Approved Drug Library Version 2 (ENZO Life Sciences) against *Staphylococcus aureus* ATCC 25923.

Compound name	Average inhibition % [1]							
	Pre-exposure				Post-exposure			
	Planktonic		Biofilm		Planktonic		Biofilm	
	Turbidity	Viability	Viability	Biomass	Turbidity	Viability	Viability	Biomass
Clindamycin·HCl	98.8	96.2	92.1	88.6	79.7	69.3	44.3	15.8
Felbamate	39.1	-13.6	-48.4	-37.6	33.8	24.1	-10.5	32.6
Cyclosporine A	40.8	-26.5	-36.2	-56.6	35.0	19.0	-10.0	6.9
Donepezil·HCl	50.1	-3.7	-24.4	6.1	36.6	32.2	22.2	47.2
Lincomycin·HCl monohydrate	99.4	97.7	99.6	96.1	96.7	96.8	96.7	3.7
Mycophenolic Acid	43.5	-49.2	-40.6	-32.6	41.4	-22.9	-0.5	39.5
Sirolimus (Rapamycin)	34.7	-12.0	14.4	-1.4	47.4	7.6	8.9	15.5
Spectinomycin·2HCl Pentahydrate	47.6	-34.2	3.8	11.8	50.5	-23.2	9.3	24.9
Amiodarone·HCl	50.1	-30.7	28.5	42.3	44.1	-5.3	4.1	35.4
Nicardipine·HCl	36.2	-52.8	2.2	8.8	40.6	1.5	-20.3	28.8
Pimozide	40.7	-35.9	-18.9	14.0	36.3	-1.3	17.6	10.8
Loperamide·HCl	47.8	-44.7	-28.7	0.5	39.4	-23.4	9.7	43.0
Tolbutamide	34.9	-38.9	-23.3	15.7	40.3	13.3	-34.2	5.0
Glipizide	31.6	-60.4	-16.2	10.0	-4.9	-24.2	-1.3	21.6
Phentolamine·HCl	41.6	-0.4	-17.5	-22.9	1.3	9.6	-31.2	-14.6
Quinine·HCl·2H2O	43.0	-43.2	-44.1	-11.4	35.4	17.3	3.2	14.8
Propafenone·HCl	61.3	18.5	-42.9	-20.4	59.1	19.2	-14.4	-17.9
Phenytoin	59.7	-51.6	-52.2	-20.6	52.3	-0.3	16.8	-17.2
Procainamide·HCl	65.7	-6.0	-44.4	-3.7	58.0	28.7	15.0	3.7
Lidocaine·HCl·H2O	59.6	-23.2	-35.5	-29.1	61.9	6.7	-11.7	-21.9
Flecainide Acetate	57.2	-10.1	-29.3	-29.8	58.0	19.1	0.8	-17.2
Rosiglitazone	53.3	-9.1	-29.8	2.9	51.4	-8.3	11.4	0.7
Amantadine·HCl	45.2	-12.9	-13.6	-0.3	63.8	25.4	-9.7	-19.4
Prazosin·HCl	52.9	-9.1	-30.3	7.1	62.7	1.1	-26.8	14.0
Clonidine·HCl	56.0	-14.3	-32.4	3.3	52.7	17.4	-33.7	-0.7
Guanabenz Acetate	55.3	-28.2	-50.5	-23.7	60.0	-3.9	-12.6	-11.2
Dihydroergotamine Mesylate	43.6	-33.5	-41.8	-5.9	36.4	-2.3	-1.4	6.0
Emtricitabine	32.8	-71.6	-8.4	-2.9	32.9	-40.0	-1.2	0.5
Betaxolol·HCl	26.8	-3.2	1.8	3.9	40.5	23.2	-19.6	-27.5
Caffeine	42.0	-37.7	-23.6	-7.1	51.0	1.2	-7.5	-11.8

(S)-Timolol Maleate	56.5	18.3	-48.7	-32.7	48.1	39.4	-15.7	-18.9
Salbutamol Hemisulfate	57.7	-23.6	-34.2	3.0	53.9	17.5	7.9	-3.0
Pindolol	66.5	11.7	-33.9	-17.2	52.9	25.4	-5.8	-22.6
Dobutamine·HCl	65.1	9.6	-39.7	-46.6	59.6	18.1	3.6	0.5
Sotalol·HCl	69.5	18.6	-25.1	-44.9	61.0	32.0	-17.3	-23.3
Maprotiline·HCl	59.6	-3.0	-12.3	-18.8	61.0	21.7	-15.3	-15.3
Pilocarpine·HCl	44.4	-9.3	-8.4	24.3	53.4	37.7	9.4	-2.9
Ipratropium·Br	56.5	-5.8	-25.4	-3.7	55.6	-0.3	-4.4	-3.1
Tropicamide	63.4	9.0	-31.1	-3.0	53.2	19.6	-28.1	-14.1
Pancuronium·2Br	64.3	-10.7	-37.5	-56.7	51.1	16.2	-53.2	-31.7
Ivermectin	67.4	30.8	-28.7	-19.3	46.2	18.9	-57.8	-38.1
Haloperidol	28.8	-71.7	-10.8	4.9	35.4	-15.7	6.6	-6.2
Cimetidine	41.1	0.0	10.8	8.5	13.2	4.8	-13.7	16.8
Zonisamide	45.4	-29.8	-27.3	-15.7	46.9	2.4	-0.2	-8.1
Zoledronic Acid Monohydrate	60.0	18.5	-19.3	-25.3	59.8	48.1	-1.8	-5.9
Naltrexone·HCl	55.0	-6.2	-21.5	-30.8	54.5	19.7	21.2	-12.9
Zolmitriptan	61.9	10.6	-30.2	-32.1	55.0	36.9	6.9	-18.7
Memantine·HCl	58.0	-14.2	-21.6	-29.0	58.0	13.7	-4.4	-23.5
Riluzole·HCl	59.4	7.2	-16.3	-45.7	59.2	31.5	1.6	3.5
Propofol	52.3	-25.0	-14.0	-9.0	58.3	16.5	3.9	-9.8
Aminophylline	52.3	-1.2	-6.9	-2.8	57.8	29.2	6.2	-8.5
Nateglinide	58.8	-3.3	-26.0	-11.0	49.9	7.1	-4.1	22.2
(±) Isoproterenol·HCl	57.9	1.8	-19.2	3.3	55.2	31.3	-14.1	-17.5
Acetylcholine Chloride	60.3	-27.0	-31.6	-48.7	51.1	11.3	-6.8	-10.8
Atropine Sulfate Monohydrate	53.8	-7.6	-20.7	-23.3	46.1	23.5	8.5	23.8
Apomorphine·HCl Hemihydrate	37.4	-70.6	9.3	18.8	24.6	-38.6	13.7	0.2
Chlorpromazine·HCl	15.0	6.6	39.8	28.8	-1.3	12.0	-4.4	8.2
Fluphenazine· 2HCl	53.2	-17.7	-29.9	-37.8	55.4	20.8	-7.4	-8.3
Risperidone	53.3	23.4	-34.6	-30.7	52.0	43.4	-1.0	3.4
Diphenhydramine·HCl	65.6	0.6	-19.3	-20.8	58.5	16.7	22.7	-30.6
Promethazine·HCl	48.8	-4.2	-10.4	11.0	47.8	34.5	29.1	7.6
Ranitidine·HCl	58.8	-24.0	-25.4	-37.3	56.8	18.4	-8.1	-5.4
L-(-)-Epinephrine-(+)-Bitartrate	63.0	18.2	-18.7	-39.6	42.3	9.8	12.0	-9.1
Norepinephrine Bitartrate Monohydrate	51.2	-22.7	3.8	-26.6	50.9	12.7	21.5	2.5
Quetiapine Fumarate	63.7	8.8	-23.6	-4.7	53.8	32.8	24.0	19.0

Imipramine·HCl	60.3	-1.7	-19.5	4.6	55.1	21.4	2.9	9.0
Amoxapine	65.1	12.9	-23.5	11.7	43.8	24.5	-4.2	17.6
Metoclopramide·HCl	65.7	-11.0	-27.2	-20.5	58.0	15.4	2.4	-7.1
Nalbuphine·HCl	55.8	-22.9	-26.4	-3.2	39.7	25.5	-18.4	-3.1
Carbachol (Carbamylcholine) Chloride	30.1	-72.8	-0.9	-4.1	41.6	-12.9	-3.0	-12.9
Famotidine	27.3	-11.0	9.7	-4.0	33.5	27.4	12.0	10.5
Isoniazid	46.8	-19.6	-7.4	-18.7	43.1	30.1	8.3	0.6
Ticlopidine·HCl	56.4	13.9	-32.1	-50.3	62.6	43.6	-1.2	0.5
Clemastine Fumarate	65.1	-12.3	-9.5	-8.8	59.1	24.2	-7.1	-5.3
Vardenafil	67.0	23.9	-25.7	-7.2	64.7	40.4	2.4	-20.9
Linezolid	98.1	96.1	87.4	79.2	93.4	95.6	87.8	-15.5
Docetaxel (Taxotere)	67.4	-7.1	-20.3	-43.6	58.1	10.1	19.3	-9.5
Olopatadine	57.5	-9.0	-7.9	-7.3	58.8	29.1	15.6	9.0
Tolcapone	69.4	35.9	-11.2	6.2	78.0	55.1	15.3	-2.6
Olmesartan	60.7	-6.2	-4.6	2.8	56.5	9.8	-1.9	-6.6
Nisoldipine	59.1	12.7	-23.4	10.5	63.3	31.2	5.4	-1.4
Olanzapine	58.1	-12.2	-16.5	-40.6	52.1	6.3	5.6	15.8
Lovastatin	57.0	4.3	-32.0	-43.8	63.3	33.1	14.5	0.0
Lamotrigine	35.3	-68.4	7.9	-29.0	30.6	-13.5	18.1	2.8
Azathioprine	50.9	-9.9	-13.2	-30.8	57.0	32.5	5.7	6.4
Sildenafil Citrate	43.1	-1.4	30.9	-3.2	14.0	14.1	7.0	17.7
Atovaquone	41.1	-37.2	-19.4	-30.4	46.4	16.3	15.4	20.5
Sertaconazole	96.1	95.1	75.3	78.1	80.1	55.2	-31.9	-7.5
Cefepime·2HCl Hydrate	96.0	96.5	98.6	86.6	96.2	97.7	97.4	-24.1
Aripiprazole	58.7	-55.7	-15.7	-49.7	52.9	-4.6	10.5	-0.6
Candesartan	61.2	15.2	2.8	-62.0	50.8	14.7	20.9	-9.8
Butenafine·HCl	56.7	-19.4	-16.6	-6.5	53.6	18.9	26.9	-11.4
Dorzolamide·HCl	60.0	20.4	-18.7	-9.6	62.3	40.6	10.2	-14.6
Escitalopram	62.1	-9.0	-9.4	-1.9	50.7	9.7	6.0	-0.3
Eprosartan Mesylate	58.6	14.8	-14.7	5.1	51.8	27.2	1.5	-1.3
Entacapone	65.6	-2.4	-17.4	-44.5	51.5	4.9	24.8	18.7
Bleomycin Sulfate	83.0	29.0	43.9	39.6	66.6	36.2	27.5	16.5
Guanfacine·HCl	62.4	-27.1	-14.3	-5.0	43.9	-15.9	26.7	-5.3
Tizanidine·HCl	22.0	-0.7	38.0	13.8	9.2	19.4	12.2	9.9
Carvedilol	52.4	-47.5	-9.7	-18.8	49.3	16.6	19.0	33.9
Flumazenil	56.8	2.6	-5.6	-48.6	65.3	46.3	1.5	5.5
Gefitinib	60.9	-37.2	-9.5	-17.5	52.7	18.7	5.7	11.2
Imatinib Mesylate	59.7	0.0	7.5	8.7	59.1	32.8	12.3	2.8

Idarubicin·HCl	99.1	96.5	74.4	67.7	90.0	92.3	66.3	-13.4
Montelukast·Na	82.4	46.6	54.4	23.3	57.0	4.2	-0.1	-32.5
Exemestane	58.0	-8.7	-12.5	-15.0	51.0	-1.7	26.1	7.7
Dinoprostone	61.0	13.9	11.6	-8.2	60.5	33.2	20.2	-5.4
Metformin·HCl	57.5	2.2	-9.0	-21.0	51.4	14.8	6.3	-7.1
Anagrelide	56.9	13.2	9.9	7.8	50.3	18.6	2.4	10.9
Dofetilide	49.1	-23.5	-16.0	-38.2	58.4	24.2	16.0	-0.5
Erlotinib	48.6	-23.1	8.1	10.7	35.7	10.6	32.4	17.3
Tacrine·HCl	31.7	-67.4	-7.5	-13.4	44.6	-1.9	11.7	5.2
Galantamine·HBr	9.1	-4.2	31.9	-3.4	22.1	9.1	15.4	-1.5
Amiloride·HCl·2H2O	34.7	-32.1	-8.5	-12.4	53.0	23.7	24.3	9.1
Amlodipine	55.5	-2.0	-21.2	-24.1	54.6	39.2	29.1	26.1
Diltiazem·HCl	59.3	-12.6	-1.0	-3.4	62.1	23.0	15.6	7.2
Nifedipine	55.4	6.1	-26.0	-9.6	49.9	25.0	17.2	5.8
Nimodipine	53.7	-51.9	-12.2	-9.9	57.7	3.1	13.1	-7.8
Verapamil·HCl	58.4	-20.1	-14.8	-45.5	53.1	24.4	9.0	-19.4
Gabapentin	38.5	-34.3	16.5	-26.4	49.6	10.6	33.3	-9.9
Felodipine	62.5	17.5	3.3	0.5	51.1	31.5	22.3	-0.7
Phenoxybenzamine·HCl	55.0	-3.7	-24.4	12.3	56.6	11.2	-1.8	-6.6
Trifluoperazine· HCl	69.6	22.2	11.2	24.1	46.7	26.0	6.2	16.6
Latanoprost	49.6	-26.6	-24.8	-43.2	49.1	10.3	24.7	15.9
Alfuzosin	52.5	-7.3	-15.4	-32.6	42.9	17.5	32.5	22.0
Bromocriptine Mesylate	40.6	-67.5	4.9	-9.8	30.5	-8.3	29.0	28.6
Clozapine	24.1	-2.4	38.0	4.7	11.2	20.0	10.5	14.5
Acitretin	49.6	39.3	-109.3	-49.9	54.9	41.4	-28.4	-22.9
Calcitriol	51.9	9.4	2.7	-17.2	54.6	43.1	17.3	1.0
Ketoconazole	59.0	-5.6	-4.4	-15.8	55.4	23.9	20.7	19.9
Cromolyn·Na (Disodium Cromoglycate)	54.9	3.4	-4.7	-4.3	56.2	37.4	17.5	-11.1
Capsaicin	60.5	-16.1	-17.6	-47.4	59.3	13.7	8.8	-42.4
Dexamethasone	62.2	8.4	6.0	-33.7	56.4	29.5	16.9	-26.0
Dipyridamole	54.1	-6.7	-8.8	-15.7	60.0	22.9	17.3	-7.6
Ethacrynic Acid	56.5	1.4	-7.7	-7.5	51.0	17.5	13.8	15.4
Indomethacin	59.5	5.0	-14.6	-1.5	49.7	4.2	11.9	8.4
Naproxen	53.7	16.2	-10.6	5.4	56.8	25.8	4.4	-3.6
Ibuprofen	56.3	-11.0	-18.3	-33.0	43.7	-3.3	38.1	26.7
Bumetanide	52.4	-4.0	-20.3	-12.5	43.6	15.2	37.2	4.2
Neomycin trisulfate	68.8	27.4	44.6	36.8	21.2	8.5	18.1	2.5
Auranofin	90.5	93.5	93.0	87.4	84.0	85.0	62.7	44.2
Captopril	44.2	-29.0	-16.9	-21.3	48.1	9.4	17.4	14.8

Tranylcypromine Hemisulfate	45.6	8.6	-10.8	-30.1	53.2	47.4	20.5	-0.8
Piroxicam	49.4	-13.9	-2.7	-30.2	59.7	37.4	-0.4	-52.8
Moxifloxacin·HCl	98.3	97.2	97.9	92.5	96.1	98.0	91.4	-2.2
S(-)-Carbidopa monohydrate	65.7	-1.9	-1.4	-32.1	51.2	18.5	17.6	-38.6
Ketoprofen	55.3	19.0	6.7	-47.6	55.1	29.3	20.7	-38.6
Meloxicam	53.7	-11.9	0.1	-7.6	58.1	18.0	19.3	-22.6
Terbinafine·HCl	47.2	-8.6	-1.5	-14.9	56.5	26.8	31.6	-14.5
Sodium Phenylbutyrate	60.7	-13.5	-13.3	1.1	48.2	-1.0	10.7	8.1
Simvastatin	48.9	1.7	-11.6	-4.6	52.4	25.3	8.1	-17.2
Goserelin Acetate	57.4	-5.1	-16.7	-37.1	49.9	10.6	26.9	12.1
Raloxifene·HCl	40.7	-9.5	24.7	-21.5	38.1	22.0	39.7	36.0
Rifampin (Rifampicin)	97.3	95.2	91.2	77.7	76.2	71.4	86.4	37.7
Etoposide	51.8	-13.9	53.2	8.9	56.4	12.0	25.2	11.2
Mitomycin C	68.5	33.0	-16.9	-49.2	50.6	26.1	36.9	12.7
Delavirdine Mesylate	44.9	13.8	-12.7	-33.8	52.8	42.6	20.0	-4.9
Daunorubicin·HCl	98.1	97.6	97.7	93.2	87.9	65.4	25.1	-6.7
Doxorubicin·HCl	68.3	-27.2	-28.1	23.5	73.9	47.4	-11.2	6.0
Cetirizine 2HCl	54.7	-1.1	3.8	-47.0	52.0	32.7	20.7	-12.7
Lapatinib Ditosylate	66.4	26.3	-6.8	-39.6	61.6	38.0	-1.2	-27.4
Pioglitazone·HCl	49.7	-11.7	-18.6	-33.0	52.2	10.3	7.8	-7.5
Rivastigmine Tartrate	53.2	7.5	-9.7	-35.4	58.5	30.6	-2.8	-16.6
Ergotamine Tartrate	51.7	-4.0	-0.3	-0.4	52.6	3.4	13.6	0.6
Sulindac	39.8	-10.2	-0.1	-2.9	50.6	13.2	18.3	-0.4
Valproate·Na	56.2	-9.7	-15.7	-27.5	53.1	11.9	26.8	22.3
Calcipotriene	45.8	-6.5	-4.3	-7.1	39.5	15.7	24.0	15.5
Zafirlukast	53.6	-25.7	29.7	-18.9	62.3	42.2	46.0	-1.0
Zileuton	15.5	4.1	46.4	24.4	14.4	9.8	30.7	21.8
Bortezomib	40.4	-13.9	-12.4	-42.5	41.9	2.6	39.9	0.3
Diazoxide	50.8	27.3	-21.4	-44.0	46.9	42.6	33.9	10.0
Glyburide	50.9	-31.9	-21.1	1.8	53.4	11.4	1.9	-8.7
Minoxidil	55.1	1.9	-3.1	-13.2	56.4	31.7	30.5	33.1
Tolazamide	61.3	-5.7	-12.0	-55.3	52.6	10.1	19.1	-11.6
Bexarotene	87.0	70.7	-29.1	-58.2	54.8	48.7	8.1	-5.8
Tranexamic Acid	50.8	-11.4	-9.4	-21.2	54.2	18.0	24.9	5.1
Celecoxib	55.0	18.3	-11.2	-26.5	66.9	50.3	4.1	-25.0
Levetiracetam	54.4	-6.5	3.2	9.8	56.8	21.4	21.0	-3.7
Letrozole	54.2	8.9	11.7	5.1	47.2	41.9	0.7	-9.8
Anastrozole	55.5	-0.7	-9.9	-27.9	54.3	29.6	-15.9	3.3

Bicalutamide	52.7	-4.4	-4.0	-28.7	36.4	29.1	12.4	30.2
Clindamycin Palmitate·HCl	99.0	94.6	85.7	88.0	84.5	79.9	61.0	12.8
Vorinostat	17.9	1.1	30.1	-11.3	33.8	18.7	21.5	2.1
Didanosine	41.7	-26.6	-20.8	-35.2	46.3	15.9	29.0	23.4
Dolasetron	50.7	24.8	-17.1	-46.3	50.1	46.6	12.8	4.6
Enalaprilat Maleate	47.8	-25.7	-3.3	-7.8	52.8	31.8	-33.8	-3.0
Fluvastatin·Na	53.4	24.1	-5.2	-9.6	58.8	58.5	2.5	-12.7
Fosinopril·Na	42.2	-15.3	-3.3	-10.1	23.2	-1.2	-4.5	-17.0
Gemcitabine·HCl	98.6	98.3	88.8	83.8	92.7	92.9	85.2	32.3
Granisetron·HCl	54.0	-10.9	-4.5	-20.3	55.4	19.7	13.7	-8.4
Oxaliplatin	57.2	17.9	5.4	-1.3	53.3	34.6	18.5	1.6
Atazanavir	50.1	-6.2	7.8	-8.2	48.3	13.9	22.0	-11.6
Mycophenolate Mofetil	43.4	-0.8	7.3	9.7	43.2	33.5	24.5	0.9
Clofarabine	44.7	-31.3	7.9	-22.7	39.7	15.2	-81.7	-27.4
Cabergoline	37.2	-12.5	6.7	8.0	43.5	19.1	18.2	1.3
Ibandronate·Na Monohydrate	62.9	-21.9	39.8	20.1	32.6	33.5	34.4	14.5
Imipenem	40.2	1.4	55.2	7.7	52.2	27.9	17.4	-17.2
Lomustine	48.5	-12.9	-24.4	-9.0	58.4	35.7	9.3	-3.5
Adapalene	41.8	23.6	-5.1	-29.8	51.9	56.8	15.5	3.5
Meropenem	4.0	6.5	-7.9	-23.4	59.3	37.7	18.8	-28.9
Oseltamivir Phosphate	48.8	17.6	-13.2	-27.7	50.1	41.6	28.3	3.2
Pamidronate Disodium Pentahydrate (Pamidronic Acid)	56.7	-31.8	-3.8	-61.7	60.7	16.8	-6.5	-23.3
Pramipexole Dihydrochloride Monohydrate	54.8	5.6	-3.0	-50.8	47.9	13.1	27.2	4.5
Triptorelin Acetate	54.2	-11.3	-13.3	-13.0	54.7	34.4	-9.5	-28.6
Risedonic Acid	48.0	9.6	-5.9	-17.5	52.5	36.5	15.6	-4.7
Rocuronium Bromide	53.0	4.8	-4.7	-10.7	55.3	20.5	14.2	-15.0
Vinorelbine	51.9	13.6	-5.4	-9.1	44.0	45.0	-19.5	-8.9
Salmeterol	60.0	6.0	-26.8	-64.5	43.1	15.1	11.8	-4.1
Vincristine Sulfate	49.4	0.1	-8.4	-21.7	42.9	24.9	-4.4	-13.1
Aspirin (Acetylsalicylic Acid)	39.5	-68.7	-5.3	-18.0	32.3	9.7	-11.8	18.0
Acyclovir (Acycloguanosine)	94.1	96.8	87.3	77.7	64.5	67.7	60.3	16.7
Zidovudine (3'-Azido-3'-Deoxythymidine)	33.8	-20.4	6.8	-13.8	41.0	9.5	21.6	-10.3

Allopurinol	96.1	97.3	74.7	53.8	94.5	98.1	93.2	-0.5
Altretamine	68.2	-23.4	27.9	13.7	52.5	27.9	16.7	6.1
Alendronate·Na Trihydrate	40.1	7.2	-19.3	-35.4	29.0	20.2	30.1	4.6
Albendazole	46.9	-10.5	-24.5	-60.6	45.6	7.1	10.3	8.1
Sumatriptan Succinate	51.6	18.0	12.9	-43.4	47.9	33.5	25.6	-28.6
Amifostine	32.8	-29.0	8.7	1.8	49.9	20.2	17.5	-8.7
4-Aminosalicylic Acid	54.2	11.9	-7.2	-27.2	28.0	46.5	25.8	5.0
Mesalamine (5-Aminosalicylic Acid)	42.7	-10.7	2.3	2.0	43.7	10.7	30.9	17.0
Ampicillin Trihydrate	45.3	10.5	18.6	14.4	52.0	32.4	12.7	-18.3
(±)-Atenolol	44.2	-30.0	-3.1	-33.5	47.4	27.3	-9.2	2.7
Atracurium Besylate	38.6	-3.6	-2.5	-38.0	40.7	31.2	-1.2	-13.6
Vinblastine Sulfate	43.9	-38.8	9.0	-26.0	34.5	0.0	-20.1	-4.2
Azithromycin	20.5	-0.6	36.7	-5.7	18.6	24.6	26.9	-12.9
Aztreonam	37.3	-28.0	3.3	-24.3	43.2	11.9	31.1	14.9
Betamethasone	39.9	17.6	-12.1	-61.5	50.2	49.3	25.8	-2.9
Bisacodyl	45.0	-4.1	-13.1	-22.9	48.1	17.5	3.8	-12.3
Buspirone·HCl	45.5	9.7	-5.1	-12.3	54.5	41.3	14.8	-13.1
Carboplatin	64.5	10.7	7.6	-34.4	53.4	25.7	28.3	-19.5
Carbamazepine	50.6	22.4	15.2	-41.0	44.8	25.8	27.4	-16.5
Cefotaxime Acid	100.3	97.0	94.6	75.4	93.0	90.2	85.2	7.6
Ceftazidime	47.7	-14.3	-5.8	1.0	50.3	13.9	10.7	-8.8
Chloramphenicol	85.8	27.8	36.1	16.1	76.2	61.1	14.1	8.7
Chlorambucil	47.2	8.8	1.1	-3.5	52.0	40.1	-10.2	-17.4
Chlorpheniramine Maleate	66.8	13.1	4.6	-23.2	50.6	27.5	3.5	-12.3
Chloroquine Diphosphate	34.7	-21.3	12.6	2.5	10.3	8.3	3.4	4.6
Thalidomide	52.3	-29.7	10.6	-22.0	10.8	-17.4	-22.0	-16.8
Ciprofloxacin	93.9	95.6	95.1	80.5	55.3	62.7	-11.5	-22.6
Citalopram·HBr	39.2	-41.0	-4.0	-19.2	39.4	-1.5	10.7	-18.1
Clarithromycin	30.1	-19.4	4.4	-37.9	50.9	46.0	-17.3	-46.4
Clomiphene Citrate	53.8	-2.4	-6.8	-30.8	53.6	34.3	15.8	-12.1
Clopidogrel Bisulfate	46.7	-0.5	-6.8	-33.2	51.7	45.9	-15.9	-12.2
Clobetasol Propionate	50.4	-1.5	1.1	-36.0	43.4	-7.7	29.9	-18.2
Orphenadrine Citrate	44.1	7.6	17.5	-55.0	55.3	45.5	-8.8	-40.7
Crotamiton	26.4	15.7	-9.9	3.3	33.8	8.2	-6.8	-1.7
Cyclophosphamide monohydrate	40.2	16.9	-24.4	20.7	21.6	8.1	-2.9	7.3
Cytarabine	29.4	26.1	-4.0	9.5	1.3	12.1	3.9	21.7
Dacarbazine	39.3	-2.6	-32.2	-22.6	25.5	-12.7	1.9	21.6
Danazol	16.8	13.8	-13.7	-9.3	-3.8	-1.2	21.8	21.4

Desloratadine	41.4	-30.1	-9.5	-21.3	49.3	11.9	-4.3	-30.1
Dextromethorphan	37.8	-4.4	13.2	-29.8	51.9	31.7	-8.4	-38.4
Diclofenac·Na Salt	38.3	-14.4	-22.1	-14.7	59.4	32.1	-6.9	-17.0
Zalcitabine (2',3'-Dideoxycytidine)	38.8	-3.6	21.2	5.1	47.0	35.7	-1.8	-37.8
Diflunisal	49.6	-5.7	-17.4	-35.9	44.0	6.9	32.2	-8.7
Disulfiram	25.4	29.4	-24.1	7.1	35.7	6.8	8.6	27.5
Doxazosin Mesylate	26.2	5.4	-26.4	20.6	13.7	-6.5	25.3	38.9
Doxycycline Monohydrate	97.5	97.3	96.7	88.4	92.2	93.0	87.4	16.2
Enalapril	44.2	-15.8	-13.6	16.0	41.6	15.8	-22.7	4.9
Esomeprazole Potassium	52.8	0.0	-35.2	-45.4	49.5	4.5	20.6	-4.8
Estradiol	40.8	-7.5	8.1	-26.6	27.4	22.0	17.9	-9.7
Estrone	39.2	-49.6	-1.9	-22.6	37.8	3.8	18.0	-11.9
Etidronate Disodium	51.9	21.7	-0.3	-18.4	20.0	36.7	-6.1	-27.9
Famciclovir	29.9	-24.3	1.8	-19.5	55.2	6.6	22.7	-11.6
Fenoldopam Mesylate	45.7	18.0	-11.6	-59.1	53.1	43.1	-36.8	-37.2
Fenoprofen calcium salt dihydrate	20.4	2.6	-23.4	16.7	33.4	9.4	12.7	11.5
Fenofibrate	29.3	0.5	-29.0	-15.2	35.5	-34.6	3.9	20.0
Finasteride	48.2	8.7	-22.9	15.3	44.3	25.0	33.9	28.3
Fluorouracil (5-Fluorouracil)	99.4	96.4	97.4	94.1	94.9	91.8	95.1	20.3
Flurbiprofen	56.5	-3.6	-23.4	-1.3	59.2	40.5	12.6	15.6
Amitriptyline·HCl	97.2	96.6	95.4	89.8	72.5	68.7	30.2	-35.0
Floxuridine	99.2	98.4	97.6	90.7	96.2	94.4	88.3	-13.2
Fluocinolone Acetonide	52.5	-7.7	16.4	-44.9	46.3	8.0	17.4	-41.6
Flutamide	64.8	38.6	-1.3	-60.6	48.2	36.4	35.0	-7.9
Fluconazole	42.5	-26.0	-2.1	-23.3	54.9	22.5	11.2	-30.4
Furosemide	31.4	2.3	-19.0	-13.9	48.6	-0.3	4.0	10.0
Ganciclovir	25.2	-7.7	-19.4	30.5	38.0	-17.7	38.4	44.3
Gatifloxacin	100.3	97.6	99.8	99.2	95.4	91.1	80.2	21.6
Gentamycin Sulfate	26.2	-20.4	9.6	13.6	42.7	-5.3	37.2	29.4
Gemfibrozil	51.7	-12.3	-19.3	-28.5	56.5	26.4	-1.6	-27.4
Glimepiride	46.0	15.3	-4.8	-14.7	47.2	39.8	3.6	-44.5
Hydrocortisone	28.2	-42.2	20.2	10.3	50.0	29.3	0.6	-19.4
Hydrocortisone Acetate	49.0	13.3	-5.1	1.9	55.2	40.4	13.0	10.5
Idoxuridine	52.3	-15.9	11.5	-8.6	43.6	19.1	9.8	3.4
Ifosfamide	24.4	3.0	23.1	-15.0	43.5	33.0	18.0	8.7
Imiquimod	22.2	-1.0	-19.2	20.1	30.7	3.2	42.9	50.3
Indapamide	17.7	-42.7	-13.0	15.7	28.7	-27.7	34.7	32.9

Itraconazole	44.5	-0.5	-3.4	7.6	52.6	14.0	38.5	15.4
Levonorgestrel	47.9	-24.1	-13.1	-19.1	59.2	-10.1	19.7	18.2
Levofloxacin·HCl	99.8	97.9	93.0	85.9	95.8	97.5	86.8	24.7
Leflunomide	39.2	-61.8	27.2	15.3	7.7	12.9	15.8	-6.9
Lisinopril·2H2O	7.8	8.9	32.5	8.6	8.4	39.2	1.7	-17.3
Loratadine	23.1	-30.5	15.0	-27.6	33.7	7.9	36.1	-1.7
Losartan Potassium	36.0	5.1	0.9	-30.4	35.5	38.1	-14.2	-11.6
Mebendazole	59.9	-44.5	-3.5	-32.4	49.2	7.1	32.7	-4.0
Medroxyprogesterone Acetate	13.0	-28.5	-21.7	23.6	29.3	-22.8	28.5	23.2
Mefenamic Acid	13.3	-45.4	-38.4	1.2	34.5	-25.1	14.0	10.7
Melphalan	46.3	-14.5	-7.1	-17.8	48.7	13.3	26.1	41.7
L-A-Methyl-Dopa Sesquihyrate	31.1	-42.8	-10.7	-0.6	46.3	-10.2	10.0	13.5
Methylprednisolone	54.3	2.1	-9.4	-6.6	54.3	19.5	23.3	33.8
Metoprolol Tartrate	46.2	-10.3	-1.1	-36.0	81.9	78.0	47.7	-14.1
Methimazole	49.7	-13.4	-2.4	-49.3	41.9	1.7	32.9	-15.9
Metronidazole	45.4	8.3	-15.2	-37.0	44.8	22.5	32.2	-22.2
Minocycline	97.2	94.4	93.9	83.5	92.7	94.9	94.6	-4.6
Mitoxantrone·2HCl	39.6	-22.3	-8.3	-18.8	55.3	13.5	16.0	-29.0
Paclitaxel (Taxol)	9.9	-22.3	-21.6	7.1	12.2	-23.8	32.0	37.9
Nabumetone	-9.9	-29.1	-22.9	-12.8	27.4	-37.0	8.4	-5.8
Naphazoline·HCl	31.9	-31.2	-19.1	-15.6	28.3	-14.1	44.2	33.6
Nefazodone·HCl	24.1	-55.3	-0.3	12.8	-26.8	-52.2	34.3	13.7
Norethindrone	35.3	-11.9	-10.7	-10.3	37.4	3.5	18.9	34.3
Norfloxacin	98.2	94.7	88.8	86.4	93.4	86.4	46.9	-10.0
Nystatin	46.2	-23.5	-1.8	4.1	45.5	17.5	12.7	-10.2
Ofloxacin	94.9	95.8	94.3	88.0	92.5	81.4	70.1	20.5
Omeprazole	35.7	-28.4	-7.6	-33.7	39.4	17.7	24.3	-4.2
Oxcarbazepine	57.2	-56.1	-2.1	-50.9	-10.7	-15.5	-26.5	-9.3
Oxiconazole Nitrate	100.1	95.6	97.3	97.2	66.8	-18.5	11.8	8.9
Oxacillin sodium salt monohydrate	99.0	97.4	86.3	86.0	91.6	71.5	63.0	35.4
Pantoprazole	49.8	-23.5	-7.7	1.8	58.5	-7.3	12.1	-2.1
Paroxetine·HCl	45.3	-26.2	11.1	17.0	47.4	8.2	43.0	19.6
Penciclovir	50.9	-17.2	-5.0	-8.0	44.1	-19.8	36.6	40.6
Pentoxifylline	21.4	-1.9	-2.0	31.5	41.5	23.7	6.4	14.8
Penicillin V Potassium	98.7	97.7	98.0	91.3	84.3	76.2	80.0	31.6
Piperacillin	95.0	94.7	71.0	71.0	67.6	80.0	51.4	27.2
Prednisolone	44.7	-7.9	-20.1	-4.0	42.5	-13.1	21.4	22.6

Progesterone	48.5	-22.8	-11.3	-9.0	48.9	9.2	29.5	40.7
Procarbazine·HCl	55.7	-27.7	-6.6	-30.5	64.1	24.2	6.6	-12.0
Prednisone	42.1	-32.5	13.3	13.3	46.5	9.2	51.2	28.7
Primaquine diphosphate	81.2	21.2	37.7	39.9	73.5	26.7	26.6	36.2
Praziquantel	51.9	-29.1	-11.4	-19.6	42.8	-2.5	15.2	34.2
Quinapril·HCl	31.0	-47.0	2.2	11.8	22.6	-17.3	25.6	23.9
Ranolazine·2HCl	56.3	-2.3	-14.4	-5.1	75.9	54.4	8.3	-3.0
Ramipril	47.9	-22.9	1.1	-19.3	45.0	16.3	33.5	31.6
Ribavirin	54.5	17.9	-15.5	-42.0	61.1	33.5	20.5	11.3
Nelfinavir Mesylate	-1.5	44.4	37.7	39.5	25.7	9.8	7.2	-3.2
Rimantadine·HCl	51.7	8.5	-16.0	-20.0	50.1	30.6	7.8	5.2
Propranolol·HCl	43.4	5.8	-4.2	15.6	49.6	21.9	25.7	27.0
Scopolamine·HBr	45.5	6.8	7.4	-34.8	62.8	35.1	21.2	-2.5
Spironolactone	40.4	-4.7	6.3	27.0	41.6	5.3	42.0	32.3
Streptomycin Sulfate	50.7	-2.3	0.5	29.8	52.2	-1.5	29.0	21.2
Sulfadiazine	37.3	-19.4	-3.1	-4.8	34.0	7.3	34.1	32.0
Sulfasalazine	45.3	-3.7	-14.6	5.0	61.8	48.7	17.7	7.3
Tamsulosin·HCl	51.0	-21.5	-14.4	-25.5	49.1	5.8	33.2	-15.0
Telmisartan	42.8	13.1	-22.5	-23.1	49.5	29.7	22.9	21.8
Terazosin·HCl	52.4	-3.5	-21.8	-9.4	47.4	-3.0	15.0	6.6
Tetracycline	51.5	-14.5	-9.9	-5.9	45.2	8.5	25.9	27.6
Temozolomide	57.8	-7.0	-4.4	-69.1	59.1	16.9	18.4	-21.3
Tinidazole	47.3	-35.0	2.2	5.2	45.9	-2.9	50.5	32.6
Tobramycin	98.5	96.2	94.7	92.8	95.7	96.5	98.6	28.7
Topotecan·HCl	46.5	-38.1	-24.8	-17.0	55.3	-5.4	31.4	26.9
Toremifene Base	90.9	33.2	46.6	66.7	34.8	-36.2	37.8	32.2
Tolmetin sodium dihydrate	53.7	1.9	-21.2	0.1	64.2	35.0	8.2	-27.4
Amoxicillin	36.9	-49.8	7.1	27.6	49.5	-3.6	42.4	26.7
Tramadol·HCl	56.4	1.9	-10.3	-54.9	67.1	34.5	11.8	-32.1
Trimethoprim	94.6	95.5	87.8	86.8	41.4	59.7	38.6	30.6
Valacyclovir·HCl	50.7	15.7	-13.8	-31.5	41.7	14.3	34.2	10.2
Vecuronium Bromide	54.5	14.5	0.4	2.6	51.3	33.2	27.3	30.8
Venlafaxine·HCl	48.2	-0.8	3.9	-22.7	56.2	18.8	6.4	-7.5
Bupivacaine·HCl	48.7	-5.3	1.9	0.0	39.0	5.0	41.6	56.2
Ketotifen Fumarate	46.7	7.2	0.7	-3.8	36.9	6.3	32.3	52.5
Naloxone·HCl	42.5	-6.3	-0.4	-4.7	29.7	8.4	26.9	25.3
Fluoxetine·HCl	43.9	-18.8	7.6	18.7	57.9	11.8	34.4	25.7
Ondansetron	51.2	-22.4	-13.7	-13.3	47.3	-10.2	26.2	32.7
Tiotropium Bromide	42.0	-7.6	-4.1	-10.1	44.5	17.4	52.1	38.5

Thioridazine·HCl	60.1	-5.5	-2.5	-27.5	32.7	-19.6	24.7	-4.3
Amrinone	47.2	-0.9	-3.1	-7.9	54.0	25.5	14.7	2.9
Milrinone	51.9	-10.3	-11.5	-41.8	64.4	13.8	5.7	-27.7
Alprostadil	37.4	-19.3	12.3	-13.3	48.3	-5.9	48.5	23.7
Misoprostol	45.3	-49.0	3.5	-5.1	43.1	-40.3	45.1	39.2
Argatroban	51.1	-27.6	3.4	-14.4	28.0	-12.3	36.4	33.4
Cilastatin·Na	28.2	-60.3	-3.2	18.2	4.6	-29.9	27.9	24.2
Butoconazole Nitrate	68.6	15.3	-4.0	-2.6	54.9	10.3	16.7	52.7
Mifepristone	47.8	-33.7	-16.1	-17.9	28.8	-25.2	29.1	35.6
Megestrol Acetate	47.0	-7.5	-14.2	-17.5	51.4	25.6	23.5	36.5
Tamoxifen Citrate	77.7	38.3	45.8	43.0	1.2	13.6	39.9	26.7
Aprepitant	58.7	2.7	-2.8	-32.7	51.5	3.3	35.5	27.2
Bosentan	38.3	-1.1	4.7	-13.0	55.1	23.0	43.2	22.9
Efavirenz	54.2	4.9	-1.9	-61.8	62.3	34.1	25.5	25.1
Miglustat (N-Butyldeoxynojirimycin·HCl)	32.3	-12.3	-1.7	-3.3	49.4	14.3	57.8	39.8
Fulvestrant	34.3	-15.9	6.5	2.4	51.3	11.6	29.2	30.3
Esmolol	41.7	-15.1	-9.6	7.8	35.7	6.7	24.3	29.4
Capecitabine	67.7	32.2	6.4	13.5	63.2	29.6	33.7	28.3
Succinylcholine Chloride·2H2O	29.8	-63.4	1.2	28.4	-3.2	2.9	23.2	40.3
Cyproheptadine·HCl Sesquihydrate	43.9	-6.4	-15.0	-20.4	42.3	18.8	4.9	22.0
Abacavir Sulfate	43.1	-13.5	-0.4	-37.6	43.5	-6.6	39.3	28.7
Acamprosate calcium	20.7	20.6	18.5	21.4	-3.0	26.4	23.0	32.5
Acarbose	39.4	-3.9	9.3	-16.7	36.8	-27.6	29.6	13.3
Acebutolol·HCl	26.8	14.0	11.0	10.3	7.9	11.0	52.6	41.0
Acetaminophen	32.8	-7.9	-6.7	-46.1	36.5	-19.9	35.0	9.0
Acetazolamide	51.9	15.5	0.4	-41.4	57.0	31.7	20.0	14.6
Acetohexamide	43.2	-6.2	4.6	-10.7	60.1	31.6	35.5	15.5
Acetohydroxamic Acid	46.6	11.9	5.8	-1.2	49.7	27.0	42.4	13.4
Acetylcysteine	52.3	-15.6	15.1	-42.6	47.0	9.1	32.9	-3.1
Acrivastine	19.2	-6.9	19.3	26.4	2.9	-1.5	31.7	41.4
Adefovir Dipivoxil	41.2	-16.6	-1.7	-5.6	60.6	31.7	32.9	30.5
Adenosine	52.4	14.2	-4.7	-50.5	51.7	23.0	32.5	24.4
Alitretinoin	64.6	56.1	-22.4	-51.4	56.5	35.2	25.8	1.3
Almotriptan malate	45.9	-16.7	5.9	7.4	54.4	22.5	31.2	18.9
Alosetron·HCl	51.9	15.5	0.4	-41.4	57.0	31.7	20.0	14.6
Ambrisentan	55.0	25.1	5.8	-20.6	43.5	47.1	-29.0	-16.9

Amcinonide	51.3	-5.4	10.1	3.4	51.6	12.4	33.3	-1.4
Amikacin Disulfate	98.8	98.6	97.1	91.4	14.5	8.8	-13.3	-12.4
Aminocaproic Acid	45.3	-22.1	1.4	-38.1	51.2	3.0	30.4	9.0
Aminohippurate·Na	50.6	-3.8	0.5	-10.9	62.3	48.1	18.3	19.6
Aminolevulinic Acid·HCl	48.1	-14.8	12.7	-33.4	57.5	17.8	18.0	-22.5
Amlexanox	45.3	2.2	-8.0	-36.0	66.6	42.9	32.5	21.5
Amphotericin B	43.3	-15.4	16.3	-27.2	58.4	17.5	28.7	-4.3
Arsenic Trioxide	53.8	14.3	12.7	-7.6	63.8	44.8	49.0	30.9
Artemether	27.2	-25.3	-11.2	-34.2	4.8	-12.3	30.0	7.7
Articaine·HCl	46.6	11.9	5.8	-1.2	49.7	27.0	42.4	13.4
L-Ascorbic Acid	52.3	-15.6	15.1	-42.6	47.0	9.1	32.9	-3.1
Asenapine Maleate	44.0	4.1	14.9	-11.5	43.0	36.1	24.1	20.8
Atomoxetine·HCl	49.2	-32.7	12.8	15.8	47.3	-1.3	55.2	48.1
Atorvastatin Calcium	48.4	-6.8	1.1	-26.3	57.5	25.6	22.6	-16.2
Azacitidine	37.5	-20.6	25.0	22.7	43.5	2.0	42.1	32.7
Azelaic Acid	45.8	8.5	5.2	-28.4	66.8	40.1	12.3	-4.4
Azelastine·HCl	40.2	-18.7	24.6	5.1	47.1	-1.5	51.0	36.4
Bacitracin	64.6	13.8	19.3	-30.6	59.4	27.6	19.0	4.1
Baclofen	37.1	-16.8	23.5	-0.2	41.5	12.2	52.8	31.3
Balsalazide	55.7	12.4	9.4	-74.4	54.6	23.3	40.0	10.2
Beclomethasone Dipropionate	35.9	-27.4	19.0	10.3	42.4	-10.2	50.5	28.9
Benazepril·HCl	46.0	-2.8	5.2	-40.5	51.4	10.1	25.7	18.8
Bendamustine·HCl	43.3	-27.8	6.6	-6.1	43.9	-24.5	43.2	48.2
Bendroflumethiazide	43.3	-9.1	5.6	-2.4	40.5	7.4	54.0	31.3
Benztropine Mesylate	39.5	-43.7	17.6	12.2	53.2	-3.1	36.6	5.6
Betaine	42.2	-8.4	13.4	1.7	42.4	22.8	46.9	33.2
Bethanechol Chloride	36.3	-44.5	5.8	-12.1	53.7	-2.5	31.7	31.2
Bimatoprost	48.3	-7.2	20.6	-15.7	41.9	16.9	47.1	40.4
Biperiden·HCl	49.9	-8.0	14.2	13.7	53.9	10.7	33.1	31.1
Bisoprolol Fumarate	39.9	-0.3	16.5	3.0	41.8	14.2	58.8	28.8
Brimonidine	39.1	-23.0	10.0	-11.0	41.9	0.6	35.5	12.7
Bromfenac	49.0	7.6	7.4	-17.6	41.5	2.0	50.5	34.4
Brompheniramine Maleate	39.4	-42.8	-3.5	-0.8	45.9	7.3	21.1	14.3
Budesonide	46.1	0.6	2.2	11.6	45.1	5.2	36.9	35.5
Bupropion	21.9	-28.8	-1.6	-15.4	32.2	-1.9	24.9	12.4
Busulfan	30.4	-11.9	0.3	23.3	34.7	15.1	53.4	46.7
Capreomycin Disulfate	23.0	-10.4	8.7	16.4	15.1	-2.6	30.7	15.8
Carbinoxamine Maleate	46.1	-39.2	11.6	17.4	43.8	-12.5	23.6	18.6
Carglumic Acid	50.4	11.9	11.5	-13.3	44.5	15.7	38.4	32.4

Carmustine	26.6	5.0	15.5	0.4	43.2	2.7	38.2	31.8
Cefaclor	83.5	7.2	40.4	38.4	75.8	22.8	9.2	21.6
Cefadroxil Monohydrate	51.0	-14.1	10.1	-6.1	33.8	2.9	40.2	26.7
Cefazolin·Na	31.6	-56.7	21.1	24.7	20.0	-12.2	30.8	39.9
Cefdinir	99.8	97.5	94.0	91.3	90.1	79.3	87.2	40.5
Cefditoren Pivoxil	95.5	72.5	85.1	81.3	80.7	40.1	58.6	41.3
Cefixime	86.0	46.2	71.1	50.4	84.0	60.0	68.4	36.7
Cefotetan Disodium	34.7	-59.5	5.0	0.7	26.8	-11.3	13.1	11.8
Cefoxitin·Na	50.8	5.0	27.5	4.1	48.1	16.9	31.9	13.7
Cefpodoxime Proxetil	77.2	9.9	58.0	18.8	66.7	15.7	50.6	38.8
Cefprozil	64.3	16.3	30.2	8.0	44.9	13.4	10.9	32.0
Ceftibuten	49.7	-0.5	20.0	-11.3	45.5	-9.3	58.9	30.3
Ceftizoxim·Na	63.6	5.1	29.8	4.0	60.5	20.8	47.0	28.4
Ceftriaxone·Na	44.4	-11.7	7.7	-47.3	49.6	13.6	32.4	-9.5
Cefuroxime Axetil	68.5	28.2	58.7	3.0	72.1	39.8	70.6	39.7
Cefuroxime·Na	56.2	-5.9	27.0	-9.1	57.8	-1.4	39.1	23.3
Cephalexin Monohydrate	51.4	-24.4	24.0	4.1	49.7	16.3	14.0	17.1
Chenodiol (Chenodeoxycholic Acid)	24.7	-26.1	-0.4	-13.8	25.4	-14.4	-4.6	16.8
Chlorhexidine Dihydrochloride	96.7	93.5	67.7	63.3	27.5	77.6	51.9	35.2
Chlorothiazide	50.0	7.0	1.1	-40.8	46.7	25.7	38.5	12.6
Chlorpropamide	46.9	-0.8	4.0	-8.1	41.4	-4.3	16.3	3.5
Chlorthalidone	24.8	7.7	9.8	13.0	-8.0	11.5	12.2	-0.3
Chlorzoxazone	38.0	-7.3	1.7	-24.1	23.7	-7.1	22.5	-2.7
Ciclesonide	62.2	58.6	4.4	7.4	-30.2	-3.3	30.7	-7.1
Ciclopirox	5.8	25.1	-0.9	-11.5	44.2	-2.6	24.2	-8.9
Cidofovir	-10.2	-5.9	4.6	5.6	-14.7	16.4	36.1	15.5
Cilostazol	44.4	-6.7	-2.2	-16.6	49.7	-4.2	33.0	1.3
Cinacalcet·HCl	46.4	14.2	21.7	-6.5	-21.9	-7.7	35.9	14.2
Cisatracurium Besylate	39.5	-6.2	15.7	-10.8	28.9	-0.7	42.0	7.0
Cisplatin (Cis-Diamineplatinum(Ii) Dichloride)	6.2	11.0	27.4	32.0	-3.4	42.5	23.1	-11.5
Cladribine	50.0	-3.2	10.2	-12.7	40.9	20.9	19.0	23.1
Clavulanate Potassium	72.2	38.4	31.8	-10.1	69.5	42.5	41.8	-24.0
Clofazimine	94.8	85.6	87.9	88.2	81.5	61.6	4.2	5.3
Clomipramine·HCl	43.7	-12.2	3.7	-8.3	44.5	16.8	19.7	18.6
Clotrimazole	88.6	28.8	5.7	-16.0	58.5	-6.0	22.1	27.8
Cloxacillin·Na	97.9	97.3	96.3	82.9	91.5	89.6	89.3	24.5

Colchicine	34.9	3.0	13.2	-20.3	33.3	7.7	37.6	28.0
Colistimethate·Na	51.9	4.2	28.3	2.1	49.7	24.5	38.2	-1.6
Colistin Sulfate	60.2	-1.4	19.5	-23.8	60.2	30.6	20.5	15.2
Cortisone Acetate	48.0	14.0	3.0	-5.3	39.9	36.7	12.6	-6.1
Cyclobenzaprine·HCl	56.1	0.8	7.7	-42.9	45.3	14.2	12.8	-23.5
Cyclopentolate	50.3	17.0	8.1	-30.3	43.1	41.0	35.5	26.9
Cycloserine	46.8	-14.1	5.3	-35.3	59.7	17.4	21.2	-31.0
Cysteamine·HCl	48.5	14.5	14.8	-10.8	41.0	34.2	24.7	26.3
Dactinomycin (Actinomycin D)	98.9	97.7	95.2	77.1	96.3	96.9	94.1	-4.2
Dalfampridine (4-Aminopyridine)	49.2	10.7	17.5	-27.1	39.9	23.4	17.2	-1.0
Dantrolene·Na	46.7	-11.9	12.3	-30.6	42.3	-4.6	23.1	12.1
Dapsone	47.8	16.0	14.1	-18.2	48.4	45.1	33.4	26.2
Daptomycin	30.9	-19.4	13.2	-5.8	41.3	-5.3	52.5	36.2
Darifenacin·HBr	52.9	3.2	5.4	-26.5	47.1	32.3	16.1	-30.1
Darunavir	44.4	-8.1	18.2	-8.6	45.4	5.2	40.3	32.8
Dasatinib	43.8	10.6	4.4	-55.7	52.6	32.9	27.7	-12.7
Decitabine	41.3	-22.1	7.9	-3.7	51.0	7.1	38.7	17.7
Deferasirox	42.6	-1.6	-8.1	-32.5	55.9	14.6	31.8	-10.7
Deferoxamine Mesylate	40.1	-6.6	29.3	12.4	66.7	33.1	39.7	27.5
Demeclocycline·HCl	98.4	96.8	93.7	78.2	95.5	95.8	79.3	-2.5
Desipramine·HCl	48.1	-3.6	19.4	-10.4	42.6	8.6	33.6	20.3
Desogestrel	51.2	-2.0	42.4	-20.0	61.4	41.3	17.2	-12.3
Desonide	36.6	-7.0	2.6	-11.8	40.1	-8.3	49.2	37.8
Desoximetasone	45.5	3.1	12.1	-27.1	40.3	25.5	37.3	20.7
Desvenlafaxine Succinate Hydrate	44.9	-8.1	7.7	-26.8	42.3	3.9	42.2	34.8
Dexchlorpheniramine Maleate	36.1	1.7	16.6	-26.3	43.4	17.1	42.0	30.4
Dexmedetomidine·HCl	38.6	-15.8	8.8	-1.9	38.6	-0.6	43.7	36.2
Dexrazoxane	39.1	-8.7	11.5	-14.9	47.3	1.3	49.8	43.0
Diatrizoate Meglumine	41.6	3.8	23.7	-13.0	42.8	16.1	45.6	25.5
Dicloxacillin·Na Salt Monohydrate	99.3	97.7	94.3	88.6	96.9	93.1	87.1	54.8
Dicyclomine·HCl	47.4	-17.7	0.3	-13.4	42.1	-15.2	32.9	38.7
Dienogest	36.2	-2.8	6.7	9.5	42.0	18.7	38.7	24.2
Difluprednate	39.0	14.8	-1.3	-8.5	39.4	15.6	44.3	37.8
Digoxin	40.9	-25.3	3.8	14.4	38.9	-1.3	45.7	34.9
Dimenhydrinate	33.6	3.4	13.4	-11.2	45.2	18.7	38.3	34.7

Disopyramide	34.5	-27.4	16.6	-3.0	37.6	3.7	42.0	27.4
Dopamine·HCl	33.3	7.7	20.8	-1.2	36.8	14.7	51.1	42.9
Doripenem	38.7	-12.3	19.7	9.2	36.2	7.3	7.5	4.1
Doxapram·HCl H2O	28.4	4.7	22.4	19.3	39.5	19.0	46.4	39.2
Doxepin·HCl	22.8	-24.4	8.0	-19.2	33.3	-11.7	29.8	27.1
Droperidol	32.3	-8.4	-2.8	12.9	40.9	12.1	30.8	21.2
Drospirenone	25.3	-24.3	4.7	17.4	0.0	-4.5	21.7	1.2
Duloxetine·HCl	37.1	-0.6	3.3	2.6	25.7	8.7	31.8	30.6
Dutasteride	36.1	-24.5	13.3	-3.1	27.8	-20.9	16.6	25.5
Dyphylline	37.0	7.3	16.8	12.9	28.0	9.5	23.9	24.7
Econazole Nitrate	96.4	66.4	47.0	42.4	44.0	-43.6	20.6	39.4
Eflornithine·HCl	35.7	-33.8	14.2	-17.4	23.7	-16.9	13.2	19.5
Epinastine·HCl	34.2	0.9	2.2	-22.3	26.2	18.2	34.3	40.1
Epirubicin·HCl	96.3	97.5	88.8	82.4	53.9	-0.6	24.6	14.6
Eplerenone	43.9	1.7	10.2	-29.4	37.1	29.0	34.8	23.8
Eptifibatide	36.2	-14.4	9.1	-45.6	41.2	-6.6	37.3	10.6
Erythromycin	98.4	97.4	95.4	82.7	84.9	86.6	68.5	2.1
Estramustine Phosphate·Na	37.1	12.2	10.1	-26.4	45.1	18.9	43.0	37.1
Estropipate	40.9	-12.7	6.1	-13.2	41.5	3.1	-2.7	8.5
Eszopiclone	27.9	-21.7	3.4	-27.3	17.2	-5.2	18.3	26.8
Ethambutol Dihydrochloride	5.1	10.2	10.6	-26.0	23.6	50.3	27.2	4.9
Ethinyl Estradiol	1.0	8.0	-11.3	-41.9	13.9	15.2	-29.7	-31.9
Ethionamide	16.3	12.4	2.8	41.8	-18.5	-1.6	-0.9	35.1
Ethosuximide	40.1	2.3	-21.0	-6.9	-6.2	27.5	22.7	38.7
Etodolac	-0.7	-8.5	-12.0	13.9	-39.1	10.5	-2.9	28.6
Etomidate	26.4	-3.2	-15.2	18.2	5.7	-26.8	14.6	27.0
Etonogestrel	6.3	5.0	-0.3	26.8	-29.2	-1.7	-1.5	33.5
Everolimus	55.8	-1.3	7.5	37.0	25.9	-6.5	23.2	30.3
Ezetimibe	15.3	-8.1	4.7	36.6	4.2	-25.6	14.3	32.3
Febuxostat	51.2	-3.0	-4.9	1.6	21.8	-11.2	7.4	26.6
Fexofenadine·HCl	11.7	-8.4	7.3	19.1	-7.2	-6.8	7.1	30.5
Fingolimod	99.2	97.8	81.2	78.9	33.3	-26.2	28.2	10.4
Flavoxate·HCl	30.3	25.6	-1.2	26.6	2.9	-17.6	1.0	39.7
Flucytosine	48.3	-3.5	-30.6	-11.4	13.3	-0.3	7.8	44.4
Fludarabine Phosphate	47.2	6.1	-12.7	6.8	20.8	-7.5	12.3	27.1
Fludrocortisone Acetate	61.2	-0.3	-26.3	-11.7	51.3	28.2	-13.8	11.8
Flunisolide	62.3	27.3	-15.3	-1.6	30.8	8.2	-1.2	9.7
Fluocinonide	65.6	19.5	-23.1	0.0	47.1	7.3	-1.5	27.1
Fluorometholone	51.5	15.9	-9.3	15.4	34.0	-3.5	12.3	10.9

Flurandrenolide	56.8	0.8	-10.4	-4.8	63.8	36.2	-5.6	-6.4
Fluticasone Propionate	65.0	-6.2	-24.3	-10.0	43.4	18.0	18.8	18.6
Fluvoxamine Maleate	44.7	6.6	-24.1	-14.1	21.9	-10.7	-18.1	13.7
Fomepizole	36.7	-8.6	-5.9	0.8	44.7	-16.6	9.9	27.9
Formoterol	63.4	16.6	-30.6	-5.9	54.6	35.5	-14.9	8.1
Foscarnet·Na (Sodium Phosphono-formate Tribasic Hexahydrate)	33.0	-0.1	-11.8	-14.7	27.3	-10.9	-14.2	12.7
Fosfomycin Calcium	87.8	64.4	19.6	-0.7	67.0	32.4	51.0	25.3
Fosphenytoin·2Na	31.8	-3.2	-14.6	7.0	22.8	-20.9	-4.7	36.4
Gemifloxacin	95.2	98.8	95.5	88.8	81.5	65.7	53.5	43.6
Glycopyrrolate Iodide	48.7	-10.6	-19.0	-9.2	47.7	19.2	-3.8	31.5
Griseofulvin	65.8	13.8	-19.4	-17.2	36.3	13.5	12.2	31.4
Guanidine·HCl	52.5	-5.6	-6.6	-6.1	51.2	17.7	0.6	-12.0
Halcinonide	43.6	6.3	-18.9	3.9	27.3	-9.0	2.5	22.5
Halobetasol Propionate	34.8	-4.6	-27.0	10.5	28.3	-16.9	-3.4	21.7
Hexachlorophene	99.6	96.3	94.7	91.5	90.3	68.4	60.5	27.1
Homatropine Methylbromide	76.0	21.7	-28.2	-8.0	34.5	-2.6	3.0	-7.8
Hydralazine·HCl	62.0	16.6	-11.3	-1.4	47.2	24.3	-13.0	-30.2
Hydrochlorothiazide	58.8	8.9	-19.1	-1.5	52.5	16.5	-1.6	-6.9
Hydroflumethiazide	51.9	10.4	-5.2	-12.7	49.9	19.0	9.4	-1.0
Hydroxocobalamin·HCl	59.2	14.1	-18.5	-9.1	49.9	15.9	-11.9	-11.1
Hydroxychloroquine Sulfate	57.1	13.6	-4.1	-11.0	48.6	28.4	-32.7	4.2
Hydroxyurea	70.9	14.6	-24.3	-30.2	50.7	2.8	6.6	14.9
Hydroxyzine Dihydrochloride	45.0	5.7	-29.0	16.2	25.8	-14.1	11.0	9.7
Ibutilide Fumarate	29.3	-21.4	-23.3	23.1	35.2	-21.8	2.3	21.6
Iloperidone	57.3	10.7	-31.5	-27.9	31.0	-3.0	-2.5	7.2
Indinavir	58.5	-25.4	-26.5	12.1	51.6	-4.2	-10.2	19.6
Irbesartan	67.8	26.6	-21.8	-24.5	40.2	6.9	2.8	-2.3
Irinotecan·HCl	58.3	0.2	-12.4	-2.8	51.6	18.3	-8.8	16.7
Isocarboxazid	57.9	19.4	-13.2	-14.4	46.3	5.2	4.9	20.5
Isosorbide Dinitrate	51.3	-2.4	-18.2	-24.4	61.2	28.7	-28.1	1.5
Isotretinoin (13-Cis-Retinoic Acid)	63.9	23.9	-11.0	-21.4	43.7	12.9	-5.0	-16.5
Isradipine	61.6	3.4	-10.5	-2.5	49.8	14.5	2.7	14.4
Kanamycin Sulfate	97.9	96.9	97.0	94.5	26.4	8.6	-44.6	-18.1
Ketorolac Tromethamine	5.7	-13.7	-2.1	3.9	7.3	-31.1	18.4	37.7

Labetalol·HCl	55.4	-1.6	-18.8	-45.7	46.6	0.1	14.4	14.8
Lacosamide	61.8	-18.3	15.7	1.8	43.1	-25.5	35.0	30.5
Lactulose	69.4	17.3	-16.6	-17.4	49.3	22.3	7.5	20.8
Lamivudine	37.1	-21.4	26.2	-14.6	39.7	-25.2	37.0	27.6
Lansoprazole	57.7	15.3	-11.8	-35.8	50.3	-0.5	5.6	23.9
Lenalidomide	37.5	-17.1	9.3	-21.3	42.0	-12.2	19.1	29.0
Leucovorin Calcium Pentahydrate	61.3	5.3	-6.8	-0.5	50.5	16.6	-1.0	12.0
Levalbuterol·HCl	42.8	-16.1	11.2	8.6	37.4	-12.0	34.1	30.0
Levobunolol·HCl	25.4	-6.8	6.6	11.0	12.9	-43.6	45.0	20.2
Levocarnitine	14.1	-19.3	-1.8	5.2	12.8	-1.6	-1.0	7.2
Levocetirizine Dihydrochloride	36.6	-4.9	8.6	-34.4	27.5	-15.3	27.1	23.6
Levothyroxine·Na	85.5	52.1	-21.4	-17.8	-0.4	-14.9	-34.9	-0.8
Lindane	44.5	2.5	12.6	-23.9	27.2	-11.6	36.3	26.5
Liothyronine·Na	30.0	-19.5	15.6	-23.2	28.7	-49.5	23.3	22.0
Lopinavir	43.7	-6.5	11.4	-30.6	43.6	-17.5	42.4	41.1
Loteprednol Etabonate	42.2	4.2	9.1	5.8	41.3	2.8	35.9	24.9
Loxapine Succinate	41.8	-33.1	12.2	-0.3	18.9	-65.5	42.7	46.3
Mafenide·HCl	7.5	-2.3	24.7	30.5	-5.1	-7.1	1.0	28.7
Malathion	55.3	14.7	1.5	13.3	30.7	-0.2	17.6	14.8
Mannitol	59.9	18.0	-9.7	-8.0	58.1	33.1	-0.7	27.9
Maraviroc	54.4	1.3	0.2	-7.5	34.8	25.4	0.7	-24.3
Mechlorethamine·HCl	48.0	9.0	-20.1	-36.8	47.1	18.8	-7.0	22.0
Meclizine Dihydrochloride	68.1	21.7	-19.4	3.2	52.0	11.4	8.7	41.0
Meclofenamate·Na	37.8	11.9	13.9	8.7	32.5	1.1	23.0	39.7
Mefloquine·HCl	70.3	-7.2	12.0	21.3	34.0	-4.7	6.5	11.6
Mepenzolate Bromide	63.8	12.6	-14.3	-7.7	62.8	21.9	-3.6	3.5
Mepivacaine·HCl	47.7	-8.7	-7.5	-4.5	50.8	25.7	-13.1	-22.5
Mequinol	48.7	-11.2	6.5	-20.0	20.8	-6.2	16.1	22.8
Mercaptopurine Hydrate	-1.6	-16.6	20.1	28.0	-14.5	-0.6	10.2	33.6
Mesna	43.3	-15.6	9.4	15.6	27.8	-25.1	30.6	2.3
Mestranol	4.5	4.3	25.6	36.8	-10.2	-16.0	9.4	31.0
Metaproterenol Hemisulfate (Orciprenaline)	14.5	17.1	8.2	23.6	26.5	-3.9	17.7	25.0
Metaraminol Bitartrate	-4.8	-8.4	27.8	33.6	-14.3	5.6	3.9	25.3
Metaxalone	48.7	-9.6	18.1	3.2	28.4	-7.0	12.6	8.2
Methacholine Chloride	5.2	6.0	45.8	35.6	-4.9	-0.9	-0.9	16.9

Methazolamide	52.6	11.9	6.0	-3.6	20.3	-3.6	7.3	22.5
Methenamine Hippurate	46.8	1.9	-5.4	11.9	33.4	-0.2	29.5	15.5
Methocarbamol	52.2	2.8	-17.0	-46.3	40.5	9.6	2.6	20.5
Methotrexate	58.7	35.3	9.1	-9.4	38.8	10.3	32.1	15.0
Methoxsalen (Xanthotoxin)	48.9	-10.8	3.5	-8.1	57.9	28.7	-6.0	13.8
Methscopolamine Bromide ((−)-Scopolamine Methyl Bromide)	55.9	19.1	11.4	17.2	31.9	9.2	17.2	-1.6
Methsuximide	51.8	6.4	-13.5	-23.1	52.4	27.9	-10.9	3.2
Methyclothiazide	45.8	15.7	10.1	1.0	33.9	22.9	10.7	2.3
Methyl Aminolevulinate·HCl	55.5	-4.5	-9.3	-11.2	59.2	31.2	-8.4	24.8
Methylergonovine Maleate	48.8	26.9	31.6	6.6	33.2	8.4	12.0	-15.6
Metolazone	58.5	10.7	-12.9	-20.3	44.5	28.1	-5.7	6.7
Metyrapone	56.6	23.8	-5.1	-46.1	48.1	32.5	-13.3	3.4
Mexiletine·HCl	54.0	13.1	-12.8	-11.3	56.2	31.4	-7.6	21.7
Micafungin	47.9	-20.6	1.5	-13.2	45.3	24.0	-8.1	-20.0
Miconazole	97.7	95.0	82.0	91.8	76.0	41.5	-15.4	24.5
Midodrine·HCl	74.2	31.1	9.4	-16.6	62.0	53.8	-7.1	20.7
Miglitol	45.9	-27.6	7.7	-4.6	43.2	15.7	5.5	-14.4
Milnacipran·HCl	55.5	21.5	11.9	-27.1	66.1	54.3	6.0	-15.4
Mirtazapine	42.8	-19.7	-3.0	-24.6	44.9	25.2	-14.1	-45.6
Mitotane	45.5	-3.1	11.5	-24.1	48.5	31.6	1.5	-18.2
Moexipril·HCl	50.2	4.4	3.3	-24.7	44.1	30.0	9.0	-6.0
Mometasone Furoate	55.5	8.9	-23.4	-19.0	41.3	1.2	5.4	3.3
Mupirocin	99.1	97.4	94.2	89.6	90.9	92.6	57.3	4.6
Nadolol	50.0	-0.3	7.6	-0.5	49.0	26.8	-5.1	-15.4
Nafcillin·Na	98.2	98.0	93.6	89.8	90.7	76.8	51.8	8.9
Naftifine·HCl	40.4	-4.7	-9.2	-14.4	47.2	20.9	8.1	-10.9
Naratriptan·HCl	47.6	-2.3	-0.7	-37.9	47.2	36.8	-22.0	-55.9
Natamycin	47.5	-3.8	-9.7	-27.2	38.3	11.7	-3.7	-24.4
Nebivolol·HCl	58.5	19.1	-4.9	-35.5	47.7	21.9	-5.3	-3.8
Nelarabine	49.5	-16.5	0.9	-17.9	43.3	4.9	8.9	30.3
Nepafenac	53.1	15.6	-13.3	-13.4	45.5	15.6	-0.6	-8.2
Nevirapine	55.9	-11.7	8.5	11.9	45.5	14.3	17.5	37.0
Niacin (Vitamin B3/ Nicotinic Acid/ Vitamin Pp)	46.8	8.6	-10.6	-3.2	51.3	20.6	-4.7	-37.7
Nicotine	55.8	-8.4	-7.9	-2.2	47.2	10.0	0.3	34.3
Nilotinib	72.0	43.0	-34.9	-14.5	62.4	42.2	2.2	-2.7

Nilutamide	49.7	-11.3	-5.0	5.2	43.7	13.2	1.5	20.4
Nitazoxanide	71.3	57.0	-3.3	-34.9	62.7	56.6	36.2	-20.9
Nitisinone	59.7	3.7	-10.2	5.3	43.6	16.3	7.9	13.1
Nitrofurantoin	60.2	13.2	-2.5	-11.6	56.4	27.0	0.7	27.2
Nizatidine	34.9	-21.3	22.4	-26.9	42.6	-18.5	48.3	43.9
Nortriptyline·HCl	56.3	5.0	1.2	3.0	44.2	9.8	-3.9	26.0
Olsalazine·Na	33.1	-28.5	18.6	12.8	43.6	-15.8	40.7	32.2
Orlistat (Tetrahydrolipstatin)	55.8	4.1	4.6	-4.6	43.5	-4.3	20.2	33.4
Oxaprozin	35.3	-20.8	11.7	22.3	44.5	-4.9	33.6	30.8
Oxtriphylline	31.4	-34.5	26.7	2.4	46.5	-7.5	20.3	24.7
Oxybutynin Chloride	54.1	20.5	0.0	-19.7	54.0	20.0	0.3	10.4
Oxytetracycline·HCl	98.7	97.1	93.1	89.3	93.4	90.8	66.3	4.7
Paliperidone	31.4	-1.0	20.8	-6.1	43.6	-0.6	42.2	34.3
Palonosetron·HCl	32.7	-38.5	25.5	-26.6	23.6	-37.6	38.2	41.2
Paromomycin Sulfate	97.1	94.7	81.6	78.6	50.4	7.4	48.4	33.7
Pazopanib·HCl	24.3	-2.3	10.4	-4.1	19.3	-24.6	14.4	32.7
Pemetrexed Disodium	10.1	-6.5	22.6	15.4	7.2	-4.8	27.1	26.0
Pemirolast Potassium	21.1	-35.0	24.8	17.0	27.3	-39.3	30.4	40.3
Penicillamine (D-Penicillamine)	54.9	18.0	33.5	22.8	62.4	27.0	38.4	27.6
Penicillin G Potassium (Benzylpenicillin)	97.5	94.2	96.6	92.9	88.8	73.1	74.5	60.7
Pentamidine Isethionate	39.0	-10.7	19.7	-10.9	43.5	-6.7	17.9	6.6
Pentostatin	34.2	-31.2	26.9	-2.6	25.1	-29.0	23.5	28.5
Perindopril Erbumine	3.2	8.6	32.0	29.0	-3.1	3.0	11.8	20.7
Permethrin	57.3	33.6	2.9	-21.6	38.0	11.6	14.5	9.6
Perphenazine	65.6	32.1	7.1	-20.4	72.7	58.3	2.0	-2.3
Phenelzine Sulfate	40.9	-1.1	2.6	-12.8	44.5	39.7	-15.1	-23.1
Phenylephrine	46.6	20.5	-1.8	-11.3	46.7	32.9	3.7	-8.2
Phytonadione	53.9	15.2	-1.4	-17.1	46.7	11.2	3.1	21.0
Pimecrolimus	32.2	-3.0	29.1	-12.4	28.3	13.1	23.2	32.2
Pitavastatin Calcium	46.0	-8.1	22.3	9.6	25.4	0.6	21.1	16.7
Podofilox	53.3	6.9	-4.9	-36.6	54.7	37.7	-2.3	22.7
Posaconazole	8.3	9.8	34.9	56.3	-13.6	5.0	5.7	-4.0
Pralidoxime Chloride	40.8	-11.2	15.5	0.1	26.0	0.7	9.8	30.3
Prasugrel	2.9	11.5	22.7	19.5	0.5	5.6	-4.3	39.9
Pravastatin·Na	42.1	0.4	8.8	4.8	31.8	-2.7	17.6	4.7
Prilocaine·HCl	35.6	-11.2	20.8	16.7	35.3	-14.9	9.6	-28.5
Primidone	-0.9	-1.3	12.2	26.8	-11.4	0.8	1.4	21.3

Probenecid	41.2	-8.8	14.2	-7.3	27.7	14.5	-8.2	-3.2
Proparacaine·HCl	38.3	6.6	15.8	0.2	21.2	-21.3	37.8	10.4
Propylthiouracil	51.7	35.7	1.7	16.3	39.8	25.2	19.3	7.6
Protriptyline·HCl	59.5	5.2	-7.5	-37.9	56.0	36.3	-21.8	-14.2
Pyrazinamide	45.2	20.9	14.7	-0.6	34.1	11.9	27.0	22.6
Pyridostigmine Bromide	50.7	2.5	-10.4	-36.7	63.9	32.0	11.1	10.0
Pyrimethamine	50.6	22.9	15.4	13.6	39.0	14.1	25.8	-18.7
Quinidine·HCl·H2O	47.3	2.7	-0.4	-6.1	63.6	47.2	12.5	6.5
Rabeprazole·Na	52.3	26.9	9.8	18.8	28.7	17.1	9.2	-1.9
Raltegravir potassium	54.6	-2.1	-5.6	-2.4	60.3	67.0	-30.5	-23.4
Ramelteon	54.5	38.2	7.7	-3.4	31.5	18.1	13.5	5.1
Rasagiline Mesylate	53.7	15.2	2.0	-26.7	44.1	17.8	26.1	14.0
Regadenoson	48.8	26.9	11.2	-8.1	65.7	53.1	8.7	7.5
Repaglinide	45.9	-12.6	-10.1	-18.9	47.8	24.5	-62.1	-54.3
Reserpine	55.0	26.7	-1.3	-42.7	64.3	51.2	0.5	2.3
Rifabutin	98.4	97.6	96.9	92.4	94.2	95.8	88.5	22.4
Rifapentine	96.1	98.9	94.9	90.6	95.5	98.5	84.9	24.2
Rifaximin	97.8	98.0	96.2	90.6	96.5	94.9	52.2	-10.0
Ritonavir	61.7	28.0	-11.8	-9.4	63.9	62.1	-26.8	-21.6
Rizatriptan Benzoate	45.8	-0.3	2.1	-15.0	50.5	27.9	-6.9	-40.9
Ropinirole·HCl	66.7	41.7	0.3	-5.1	58.4	38.4	12.5	8.0
Ropivacaine·HCl Monohydrate	47.1	-12.7	16.2	-21.7	49.6	28.9	2.3	-12.8
Rosuvastatin Calcium	53.8	5.4	2.9	-13.6	57.3	49.9	-1.7	-42.5
Rufinamide	46.0	-6.7	3.9	-28.8	49.6	29.7	-26.8	-23.7
Saquinavir Mesylate	51.7	6.6	-4.0	-4.4	90.7	80.3	23.3	5.2
Selegiline·HCl	53.1	-21.0	-13.7	-25.2	44.9	11.6	0.5	-11.4
Sertraline·HCl	99.6	98.2	98.3	95.9	54.7	24.2	-21.1	-33.7
Silver Sulfadiazine	40.5	1.5	7.6	-2.4	49.5	31.7	4.1	-27.8
Sitagliptin Phosphate	45.2	11.2	6.7	-45.4	50.2	40.1	-25.2	-53.2
Sorafenib Tosylate	72.8	15.3	36.1	24.8	58.3	27.4	1.2	-19.3
Stavudine	47.4	16.9	-5.0	-15.5	48.5	40.3	-1.5	-11.1
Streptozocin	58.5	7.1	5.8	-26.6	58.3	33.7	1.7	-9.0
Sulconazole Nitrate	98.0	93.1	64.9	60.6	76.1	56.6	6.9	-22.3
Sulfacetamide·Na	55.2	1.8	4.9	-12.9	55.8	23.9	-10.7	-16.2
Sulfamethoxazole	63.4	18.5	-4.9	-27.5	52.2	41.9	12.0	-10.4
Sulfanilamide	55.5	5.8	-7.3	12.9	51.4	19.5	18.7	24.3
Sunitinib Malate	42.4	16.9	-15.2	-18.5	59.1	45.6	-11.5	-36.4
Tacrolimus (Fk506)	56.1	-6.2	-9.3	-1.5	53.8	15.6	4.5	25.7
Tadalafil	43.6	18.8	-2.2	-17.3	40.9	19.0	-7.9	-7.3

Tazarotene	48.5	-9.4	-12.9	9.4	46.8	5.2	0.7	22.9
Telbivudine	37.6	31.5	3.0	-20.0	48.9	39.1	-27.6	-20.1
Telithromycin	98.5	96.8	91.6	88.0	95.7	97.0	94.1	43.7
Temsirolimus	31.9	-8.7	31.6	-0.2	40.6	-5.0	20.8	9.8
Teniposide	98.1	97.1	85.6	86.8	87.6	71.3	38.0	26.3
Tenofovir	37.9	-25.8	32.7	8.5	44.9	7.8	24.4	13.0
Terbutaline Hemisulfate	55.0	-2.8	0.3	-13.4	45.4	5.9	6.3	13.8
Terconazole	24.5	-15.4	28.4	12.6	48.1	8.7	25.4	15.4
Testosterone Enanthate	48.9	2.2	-5.2	10.0	58.4	29.3	5.4	15.8
Tetrabenazine	30.7	-17.0	23.6	14.5	55.7	11.7	3.9	13.9
Tetrahydrozoline·HCl	57.8	4.5	-7.1	-17.9	73.6	48.3	3.0	28.7
Theophylline	32.3	-7.6	23.5	0.7	41.0	5.5	16.8	15.2
Thioguanine (6-Thioguanine)	30.9	5.3	22.0	-8.8	39.1	16.8	19.7	20.1
Thiotepa	32.7	-21.1	18.2	0.8	19.2	-33.5	26.9	24.3
Tiagabine·HCl	32.0	-20.8	19.9	34.8	22.2	-17.2	24.5	19.3
Tigecycline	39.8	5.1	28.3	10.8	45.6	20.3	-7.3	8.7
Tiludronate Disodium	0.5	-17.4	10.3	28.6	-25.7	-20.8	4.9	15.0
Tiopronin	9.3	0.8	11.4	17.7	7.6	-5.1	15.1	16.8
Tirofiban·HCl monohydrate	16.9	-20.4	28.1	20.1	24.1	-14.0	11.0	4.5
Tolterodine Tartrate	26.9	10.2	21.4	-11.1	41.3	23.8	-16.1	-0.2
Tolvaptan	20.5	-19.7	19.6	14.4	30.8	-22.6	41.1	27.1
Topiramate	-0.4	9.8	18.2	32.1	-26.5	19.1	-22.1	-4.1
Torsemide	54.4	34.5	-0.1	1.0	28.8	6.0	21.8	23.1
Trandolapril	57.2	35.7	-12.2	-22.6	59.0	48.0	-4.0	-17.0
Travoprost	41.9	12.9	14.8	-19.3	50.8	39.3	-4.0	-25.6
Trazodone·HCl	43.4	24.4	0.7	-27.8	45.6	43.4	-13.8	-19.6
Tretinoin	54.4	18.3	-7.3	-15.4	51.3	27.8	3.6	12.6
Triamcinolone Acetonide	36.6	4.8	12.3	-8.6	39.6	13.6	15.3	10.6
Triamterene	48.3	1.6	8.6	-1.5	21.3	-24.3	39.4	1.6
Trientine Dihydrochloride	52.7	13.5	-6.8	-23.2	51.5	20.4	11.5	-1.9
Trihexyphenidyl·HCl	-4.1	13.2	25.1	26.7	-10.3	8.9	-0.6	-7.8
Trimethadione	44.2	34.0	5.0	14.4	20.9	9.2	38.2	20.4
Trimethobenzamide·HCl	58.4	31.5	3.4	2.8	67.5	60.6	-0.4	-19.1
Trimipramine Maleate	51.9	17.1	12.1	-6.0	57.8	50.8	9.7	-30.7
Trospium Chloride	38.5	22.8	-4.5	-7.6	47.8	44.5	-2.6	-24.1
Ursodiol	59.2	19.7	3.2	-19.4	55.5	52.9	3.8	5.7
Valganciclovir·HCl	37.0	5.2	19.8	3.8	41.6	22.6	10.8	3.9
Valsartan	-12.9	8.1	30.7	22.5	-24.0	46.7	-21.9	-25.9

Vancomycin·HCl	96.9	96.6	78.9	70.1	91.9	95.2	85.4	12.2
Varenicline Tartrate	43.5	-4.1	20.8	11.9	15.5	-16.3	41.9	26.7
Vigabatrin	46.6	1.4	-7.8	-24.2	65.1	49.1	-0.6	-3.4
Voriconazole	20.2	5.2	-3.3	-3.5	13.7	-7.3	5.2	-6.3
Warfarin·Na	40.8	1.7	6.0	-12.8	49.2	19.7	3.6	1.6
Zaleplon	51.4	-5.8	5.2	-17.7	53.3	38.0	-17.6	8.4
Zanamivir	28.3	-15.4	21.0	2.3	45.3	12.3	7.1	-3.7
Ziprasidone hydrochloride	19.0	-8.5	19.9	-2.9	11.6	-8.1	-24.6	9.9

[1] Results are averages from two separate experiments.
