# Supplementary material for: Screening of FDA-Approved Drugs Using a 384-Well Plate-Based Biofilm Platform: The Case of Fingolimod
Source: Microorganisms. 2020 Nov 21;8(11):1834. doi: 10.3390/microorganisms8111834 (PMC7700524; doi:10.3390/microorganisms8111834)
Supplement: Supplementary file 1 [file microorganisms-08-01834-s001.zip › Table S2.pdf]

Supplementary table 2. Compounds and results of the activity confirmation of the initial pre-exposure hits from the Screen-Well FDA Approved Drug Library Version 2 (ENZO Life Sciences) at 10 µM against *Staphylococcus aureus* ATCC 25923.

**Compound name**	**Average inhibition %** [1]							
	Pre-exposure				Post-exposure			
	Planktonic		Biofilm		Planktonic		Biofilm	
	Turbidity	Viability	Viability	Biomass	Turbidity	Viability	Viability	Biomass
Clindamycin·HCl	96.3	94.6	98.1	100.0	71.8	65.4	39.2	11.0
Lincomycin·HCl monohydrate	99.4	99.2	100.7	97.2	61.5	75.8	62.2	20.8
Linezolid	96.7	97.4	99.1	95.6	71.3	65.2	5.6	19.8
Sertaconazole	99.8	98.9	99.2	99.7	38.4	-41.3	21.3	38.0
Cefepime·2HCl Hydrate	99.7	98.7	102.0	94.5	91.1	78.0	76.1	49.7
Idarubicin·HCl	99.3	98.9	101.8	98.4	57.5	-19.5	-78.7	2.2
Auranofin	98.2	99.2	100.3	95.6	90.4	86.0	66.9	42.2
Moxifloxacin·HCl	98.5	99.3	100.4	98.7	86.7	81.2	77.3	36.5
Rifampin (Rifampicin)	98.7	99.8	101.3	96.0	77.5	83.5	74.8	40.8
Daunorubicin·HCl	95.3	97.5	97.4	88.4	39.6	-21.1	-57.9	-7.6
Clindamycin Palmitate·HCl	95.0	95.1	96.6	99.3	77.5	76.7	37.4	-6.1
Gemcitabine·HCl	98.4	99.0	98.7	100.0	79.8	56.7	24.7	3.8
Acyclovir (Acycloguanosine) Zovirax	95.2	95.2	83.1	86.2	34.8	21.5	-5.1	2.3
Allopurinol	7.9	7.0	14.1	11.9	89.4	85.1	37.1	-11.7
Cefotaxime Acid	96.8	96.5	91.6	94.7	35.4	40.6	50.5	4.7
Ciprofloxacin	94.2	98.0	98.2	94.6	57.6	56.0	3.4	-10.6
Doxycycline Monohydrate	96.5	98.8	99.5	97.9	78.6	78.6	19.9	-6.2
Fluorouracil (5-Fluorouracil)	96.4	98.0	96.5	97.3	64.6	63.8	29.3	0.0
Amitriptyline·HCl	7.9	4.9	17.8	13.5	38.4	26.1	-29.9	-7.8
Floxuridine	96.5	98.5	98.5	98.2	64.4	57.0	5.6	3.8
Gatifloxacin	96.9	98.8	96.5	96.6	75.9	71.2	58.0	20.8
Levofloxacin·HCl	89.1	94.1	95.4	94.3	69.3	63.4	60.3	26.2
Minocycline	96.1	96.7	98.6	95.7	73.3	85.1	51.9	-10.7
Norfloxacin	79.2	50.2	62.2	59.6	76.2	66.1	21.0	-1.7
Ofloxacin	91.9	95.2	98.1	98.3	63.1	44.3	34.2	-2.9
Oxiconazole Nitrate	97.8	96.6	94.5	95.9	24.2	-44.6	-18.6	-7.5
Oxacillin sodium salt monohydrate	98.2	97.8	96.6	96.4	62.4	17.6	49.7	8.0
Penicillin V Potassium	96.4	98.8	99.8	95.6	82.1	71.1	77.8	12.8

Piperacillin	98.1	98.8	100.6	101.3	77.0	60.5	70.1	32.8
Tobramycin	96.5	96.8	92.4	94.6	54.1	38.7	5.6	6.1
Trimethoprim	97.9	95.9	93.5	95.6	20.3	33.8	-11.8	-7.8
Amikacin Disulfate	97.9	98.8	99.6	97.3	1.5	-3.9	17.1	5.6
Cefdinir	96.2	97.5	98.4	96.2	86.9	82.4	87.3	52.8
Cefditoren Pivoxil	95.6	88.4	99.4	96.5	84.1	76.1	70.1	10.0
Clofazimine	96.4	98.6	97.9	97.8	33.3	-22.1	-40.7	-6.1
Cloxacillin·Na	96.7	97.6	99.9	98.2	85.0	75.4	84.9	10.2
Dactinomycin (Actinomycin D)	98.1	97.2	96.9	94.3	90.7	80.6	54.6	12.5
Demeclocycline·HCl	96.9	97.4	99.5	98.8	88.6	91.4	55.6	-9.1
Dicloxacillin·Na Salt Monohydrate	97.9	98.1	100.2	98.0	79.8	43.3	65.2	17.4
Econazole Nitrate	93.9	86.0	90.8	92.9	3.0	-69.2	6.8	19.0
Epirubicin·HCl	72.5	5.3	61.4	68.0	23.3	-6.7	-9.6	9.9
Erythromycin	97.9	98.1	92.8	86.3	63.3	71.8	38.8	3.3
Fingolimod	98.2	98.5	100.5	96.6	35.6	13.9	-14.2	-8.7
Gemifloxacin	95.9	98.9	100.9	45.2	55.5	77.4	52.8	-3.9
Hexachlorophene	91.8	93.2	94.2	43.0	67.0	60.0	73.3	30.5
Kanamycin Sulfate	96.2	96.2	92.2	94.1	35.5	14.3	5.6	-7.7
Miconazole	96.4	98.4	97.9	95.0	10.0	-50.5	8.6	28.6
Mupirocin	96.0	98.3	100.1	98.3	84.5	87.6	59.5	10.1
Nafcillin·Na	95.6	95.0	88.5	90.4	55.0	69.4	77.8	20.7
Oxytetracycline·HCl	95.7	98.4	99.9	39.1	93.6	94.7	50.6	-10.8
Paromomycin Sulfate	97.7	98.8	97.5	98.0	25.3	9.8	0.5	15.6
Penicillin G Potassium	90.9	94.9	97.0	96.9	67.2	61.9	66.2	40.7
Rifabutin	95.6	99.4	99.7	100.4	83.6	81.6	60.9	17.5
Rifapentine	96.7	99.4	100.2	96.3	84.4	81.3	55.5	-7.5
Rifaximin	97.2	97.2	94.3	93.0	51.3	74.8	47.5	-1.6
Sertraline·HCl	17.0	-13.3	9.4	19.7	5.6	-15.9	7.7	15.3
Sulconazole Nitrate	98.6	99.0	98.9	98.7	34.8	-20.4	-19.1	-7.5
Telithromycin	96.4	97.6	99.5	96.3	93.4	93.0	37.0	-9.3
Teniposide	97.1	97.7	68.4	76.1	28.3	2.3	17.9	4.1
Vancomycin·HCl	96.4	99.5	100.7	99.3	65.0	47.5	35.2	-7.3

[1] Results are averages from two biofilm replicates.
