# Supplementary figures and images for: Screening of FDA-Approved Drugs Using a 384-Well Plate-Based Biofilm Platform: The Case of Fingolimod
Source: Microorganisms. 2020 Nov 21;8(11):1834. doi: 10.3390/microorganisms8111834 (PMC7700524; doi:10.3390/microorganisms8111834)

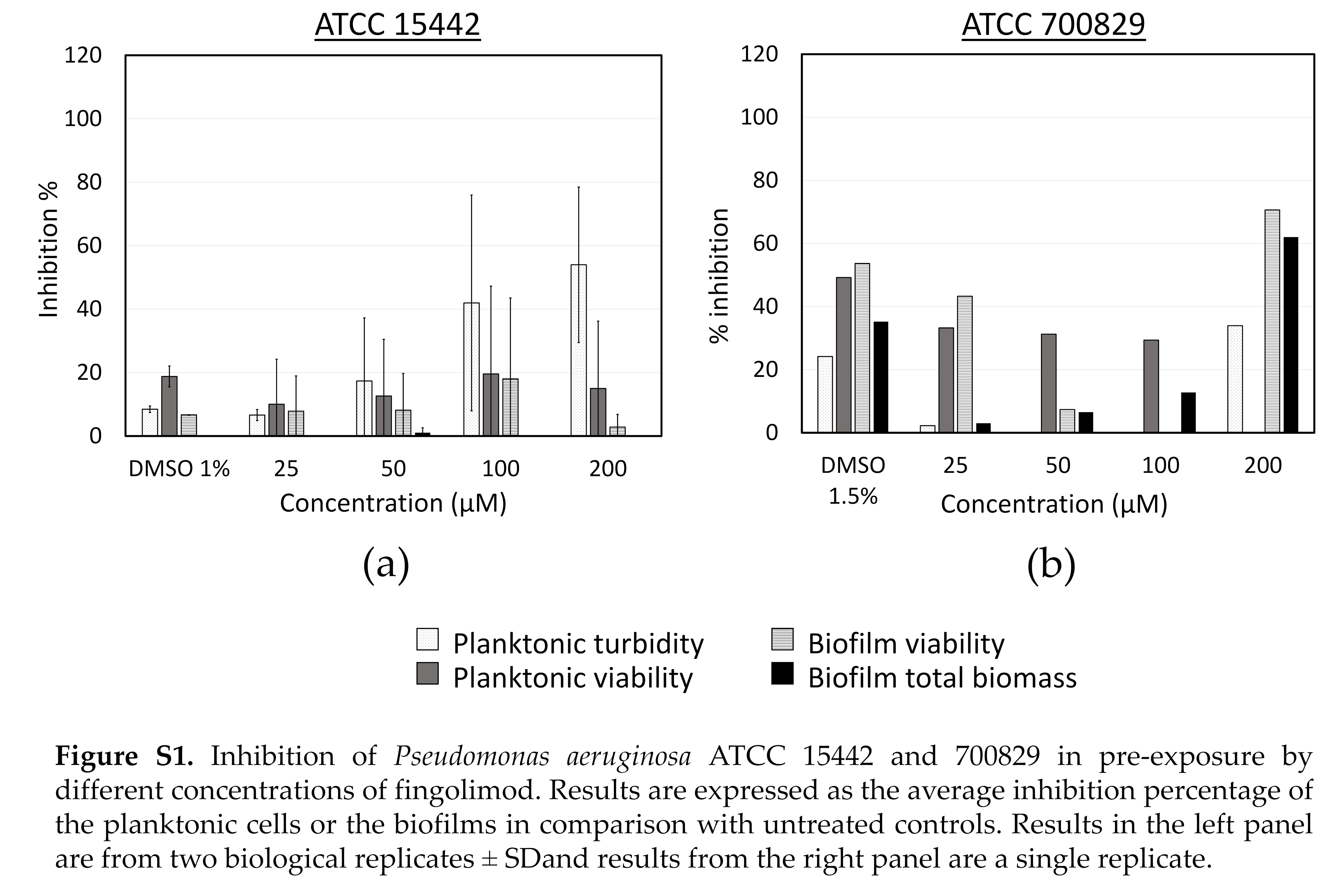

Supplement: Supplementary file 1 [file microorganisms-08-01834-s001.zip › Figure S1.jpg]
